# Ancient DNA from the Asiatic Wild Dog (*Cuon alpinus*) from Europe

**DOI:** 10.3390/genes12020144

**Published:** 2021-01-22

**Authors:** Ulrike H. Taron, Johanna L. A. Paijmans, Axel Barlow, Michaela Preick, Arati Iyengar, Virgil Drăgușin, Ștefan Vasile, Adrian Marciszak, Martina Roblíčková, Michael Hofreiter

**Affiliations:** 1 Institute for Biochemistry and Biology, University of Potsdam, Karl-Liebknecht-Str. 24–25, 14476 Potsdam, Germany; paijmans.jla@gmail.com (J.L.A.P.); axel.barlow.ab@gmail.com (A.B.); michaela.preick@uni-potsdam.de (M.P.); michael.hofreiter@uni-potsdam.de (M.H.); 2 Department of Genetics and Genome Biology, University of Leicester, Leicester LE1 7RH, UK; 3 School of Science and Technology, Nottingham Trent University, Clifton Lane, Nottingham NG11 8NS, UK; 4 Department of Biological Sciences, University at Albany, 1400 Washington Avenue, Albany, NY 12222, USA; aiyengar@albany.edu; 5 Emil Racoviţă Institute of Speleology, Romanian Academy, 31 Frumoasă Street, 010986 Bucharest, Romania; virgil.dragusin@iser.ro; 6 Research Institute of the University of Bucharest, Earth, Environmental and Life Sciences Division, Panduri 90–92, 050663 Bucharest, Romania; 7 Department of Geology, Faculty of Geology and Geophysics, University of Bucharest, 1 Nicolae Bălcescu Avenue, 010041 Bucharest, Romania; yokozuna_uz@yahoo.com; 8 Department of Paleozoology, Faculty of Biological Sciences, University of Wrocław, Sienkiewicza 21, 50-335 Wrocław, Poland; adrian.marciszak@uwr.edu.pl; 9 Moravian Museum, Anthropos Institute, Zelný trh 6, 65937 Brno, Czech Republic; mroblickova@mzm.cz

**Keywords:** *Cuon alpinus*, dhole, ancient DNA, mitogenome, hybridisation capture, canids

## Abstract

The Asiatic wild dog (*Cuon alpinus*), restricted today largely to South and Southeast Asia, was widespread throughout Eurasia and even reached North America during the Pleistocene. Like many other species, it suffered from a huge range loss towards the end of the Pleistocene and went extinct in most of its former distribution. The fossil record of the dhole is scattered and the identification of fossils can be complicated by an overlap in size and a high morphological similarity between dholes and other canid species. We generated almost complete mitochondrial genomes for six putative dhole fossils from Europe. By using three lines of evidence, i.e., the number of reads mapping to various canid mitochondrial genomes, the evaluation and quantification of the mapping evenness along the reference genomes and phylogenetic analysis, we were able to identify two out of six samples as dhole, whereas four samples represent wolf fossils. This highlights the contribution genetic data can make when trying to identify the species affiliation of fossil specimens. The ancient dhole sequences are highly divergent when compared to modern dhole sequences, but the scarcity of dhole data for comparison impedes a more extensive analysis.

## 1. Introduction

Many species’ common names are derived from their modern distribution, although this may not reflect the full range of a species’ former distribution, as in the case of the Tasmanian devil [1]. In fact, during the Pleistocene, many species had a much wider geographical distribution than they do today, for example, leopards [2] and spotted hyenas [3], both of which ranged across all of Eurasia and Africa prior to the last glacial maximum. Fossil findings of the Asiatic wild dog, also referred to as dhole or cuon, also indicate a former occurrence throughout both Asia and Europe, and even extending to parts of North America (e.g., [3,4,5,6,7,8,9,10,11]). The dhole is assumed to have gone extinct from most of its Pleistocene distribution at the end of the Pleistocene or the beginning of the Holocene [3,8]. Today, dholes are restricted to South and Southeast Asia [12,13,14]. Due to its recent, continuous population decline, the International Union for Conservation of Nature and Natural Resources (IUCN) has listed the species as endangered since 2004 [13,14], although its recent range contraction is small compared to the huge range loss the species suffered at the end of the Pleistocene. 

Phylogenetic analyses of mitochondrial genomes, as well as of extensive nuclear data, place the dhole as a sister lineage to a clade containing wolves, dogs, coyotes and jackals (e.g., [15,16,17]), diverging approximately 5.22–7.06 million years ago [18]. While its phylogenetic position within canids is well resolved, there is still a lot of uncertainty on subspecies taxonomy and phylogeographic structure of the dhole, as genetic data are only available for few individuals (e.g., [15,16,18,19,20,21,22]). Based on fur colour and fur length, up to eleven subspecies have been defined for extant populations (e.g., [12,13]), although genetic support was only found for two major phylogeographical groupings throughout Southeast Asia [21]. For ancient dholes, additional fossil species or subspecies have been proposed, based on morphological characteristics ([3,4,10] and references therein). How these ancient populations relate to modern Asian populations is unknown to date. 

The identification of ancient dhole remains is complicated, as fossil remains from dholes are both rare and difficult to distinguish from those of other canids. Although dholes are generally smaller and more slender than co-distributed canids (i.e., wolves and golden jackals), there is some overlap in dimensions [7,10,23]. Furthermore, Pleistocene dholes were larger than their living relatives [10,24], making a reliable identification of fossils even more challenging. The analysis of ancient DNA from subfossil dhole remains can help mitigate the challenges associated with the identification of ancient remains, thus providing independent evidence for species assignment. 

In this study, we analyse ancient DNA sequences from several (putative) dhole specimens, which have been identified based on morphological traits. We are able to identify the remains at species level with high confidence. To our knowledge, these data represent the first genetic data from Pleistocene dholes, allowing for an initial insight into the genetic structure and diversity that was lost when the dhole disappeared from much of its range at the end of the Pleistocene.

## 2. Materials and Methods 

### 2.1. Sample Description

Our dataset encompasses six putative ancient dhole samples, and one modern sample. The ancient samples originate from the Czech Republic, Bulgaria, and Romania (one site each). Ancient samples have been identified as dhole (*Cuon alpinus* or *Cuon alpinus europaeus*) based on morphological characteristics of the bones analysed or bones that had been found in close proximity and were assumed to originate from the same animal [25,26,27]. The modern sample was taken from a captive individual (Allwetterzoo Münster, Germany) for which a 246 bp long piece of the control region has previously been sequenced (AY682716, [21]). Detailed sample information is provided in Table 1.

### 2.2. Mitochondrial DNA Sequencing 

#### 2.2.1. Modern Sample

DNA for the modern sample was extracted using a QIAamp DNA blood mini kit following the manufacturer’s instructions. First, this high-molecular-weight DNA extract (average fragment size: ~50 kbp, concentration: ~1.4 ng/µL, both determined on an Agilent Tapestation 2200) was used as input for long range polymerase chain reactions (LR PCRs) to amplify the complete mitochondrial genome. For this purpose, three primer pairs (Table S2) were designed using the web interface of Primer3Plus [29] using a dhole mitochondrial genome sequence (GenBank accession number: NC_013445.1) as reference sequence. The three overlapping fragments (5751, 6486 and 8056 bp in length) cover the entire mitochondrial genome. LR PCRs were carried out in a total reaction volume of 50 µL containing 1× TaKaRa LA buffer (10×), 0.4 mM dNTPs (2.5 mM), 0.05 U/µL TaKaRa LA polymerase (TaKaRa Bio Inc., Cat No. RR002A, Shiga, Japan) and 0.4 µM of each primer (10 µM), using 1 µL DNA extract as starting template. Cycling conditions included an initial denaturation at 94 °C for 3 min, followed by 35 cycles of 94 °C for 15 s, 62 °C for 30 s, and 72 °C for 10 min. After cycling finished, the reaction was completed by a final extension of 72 °C for 10 min. LR PCR products were purified using the QIAquick purification kit (Qiagen, Cat No. 28104, Hilden, Germany) following the manufacturer’s instructions. Amplification success and product size were confirmed on a 1% agarose gel stained with SYBR Safe (Thermo Fisher Scientific, Cat.-No. S33102, Waltham, MA, USA). After pooling the LR PCR products in equimolar amounts, they were sheared to an average fragment length of 150 bp using a Covaris S220 following the manufacturer’s instructions. Successful shearing was verified on the Agilent Tapestation 2200. Sheared LR PCR fragments served as input to build a double-stranded, double indexed DNA (dsDNA) library following [30] with slight changes. Instead of 20 µL DNA extract, only 10 µL of sheared LR PCR products were used as template for the blunt-end repair reaction. The adapter fill-in reaction was incubated at 37 °C (instead of 25 °C) for 20 min followed by 80 °C for 20 min and put on hold at 12 °C. In the amplification and indexing reaction, Herculase II Fusion was used instead of AccuPrime Pfx DNA polymerase. The reaction was set up as follows: 11.4 µL water, 16 µL Herculase buffer (5×), 0.8 µL dNTPs (25 mM each), 0.8 µL Herculase II Fusion (5 U/µL), 6 µL P5 primer (10 µM), 6 µL P7 primer (10 µM) and 39 µL product of the adapter fill-in reaction. The reaction was incubated in a thermal cycler using the following cycling conditions: 2 min at 94 °C, followed by the predetermined number of cycles (via qPCR) of 30 s at 94 °C, 45 s at 60 °C and 45 s at 72 °C, followed by a final extension for 3 min at 72 °C, and put on hold at 12 °C.

#### 2.2.2. Ancient Samples

DNA of six ancient samples was extracted using 15–54 mg of bone powder following a protocol specifically adapted for the recovery of short DNA fragments from ancient samples [31]. Negative controls were included in all laboratory experiments. A total of 20 µL of ancient DNA extract was used to prepare single-stranded, double-indexed DNA (ssDNA) libraries ([32] for Romanian samples; [33] for other ancient samples). Samples were processed in dedicated laboratories with appropriate contamination precautions in place, e.g., modern and ancient samples were processed in separate laboratories at different times, and rooms and equipment dedicated to the treatment of ancient samples were decontaminated regularly.

#### 2.2.3. Capture

SsDNA libraries of ancient samples were enriched for mitochondrial reads by performing two consecutive rounds of in-solution capture, following [34], with slight modifications. First, sonicated LR PCR products were built into bait libraries. Blunt-end repair reactions were set up in a smaller reaction volume of 35 µL using a final concentration of 100 µM (each) for dNTPs and of 0.1 U/µL for T4 DNA polymerase. Adapter ligation reactions were set up in a total volume of 60 µL using 35 µL of the blunt-end reaction products as template and a final concentration of 6.25 µM (each) of the adapter mixture. Reaction products were purified using MinElute columns (Qiagen, cat. no. 28004) following the manufacturer’s instructions, performing two consecutive elution steps using 10 µL EB buffer each time. When setting up hybridisation reactions, human COT-1 was omitted and the corresponding volume was replaced with water. Hybridisation bait and library mixture was incubated in a PCR machine at 95 °C for 5 min, cooled down to 65 °C at 0.1 °C/sec and incubated overnight at 65 °C for ~24 h. For the following immobilisation and washing steps, 6 µL of pre-washed streptavidin-coated beads (Dynabeads MyOne C1, Thermo Fisher Scientific, cat. No. 65001) were added to each sample. Beads and hybridisation reactions were incubated under constant rotation at 22 °C for 20 min. Afterwards, beads were washed multiple times with BWT, HW (1× PCR GOLD buffer (10×), 2.5 mM MgCl_2_ (25 mM)) and finally with TET buffer. The final amplification reaction was carried out in a total reaction volume of 60 µL using 30 µL of the purified capture product as template. The following PCR conditions were applied: initial denaturation at 95 °C for 2 min followed by the predetermined number of cycles (via qPCR) at 95 °C for 30 s, 60 °C for 45 s, 72 °C for 45 s and, after cycling, a final extension at 72 °C for 3 min. Amplified products were purified using the Qiagen MinElute kit following the manufacturer’s instructions using two times 10 µL of EB buffer for the final elution.

#### 2.2.4. Sequencing

All libraries were sequenced on an Illumina Nextseq 500 machine, using custom primers to sequence read 1 and the index of read 2 [32,35]. Ancient samples were sequenced prior to (either 75 bp single-end or paired-end reads) and after capture (75 bp single-end reads). For the modern sample, 150 bp paired-end reads were generated.

### 2.3. Data Processing

Illumina-specific adapter sequences were trimmed from all raw reads using cutadapt (v1.12; [36]) with default settings and reads shorter than 30 bp were discarded. For the modern sample, paired-end reads were merged using flash (v1.2.10; [37]) using a maximum overlap (-M) of 150 bp. Since more than 90% of the reads could be merged, only merged reads were used for mapping. For an evaluation of contamination, pre-processed reads were run through a BLAST [38] search (blastn 2.8.1+; nt database, last update 20th March 2019). 

Pre-processed reads were mapped, parsed, filtered for reads with low mapping quality (MAPQ < 30) and duplicates removed using BWA aln v.0.7.8 and samtools v0.1.19 with default settings [39,40]. Ten canid mitochondrial genomes and a human mitochondrial genome were used as references (Table S3) for read mapping. Basic statistics on mapping results were extracted using samtools idxstats and samtools depth [40]. 

Published data for a modern gray wolf were downloaded from the Sequence Read Archive (SRA accession number: SRR2149873; [41]). Adapter trimming and merging was performed as described above. A random subset of ten million (unmerged) read pairs was created using seqtk (v1.2; available from https://github.com/lh3/seqtk). Data were treated as described above, except for the duplicate removal, for which Picard’s MarkDuplicates tool (v1.111; “Picard Toolkit” GitHub Repository, http://broadinstitute.github.io/picard/; Broad Institute) was used.

As a means to quantify how uniform reads were distributed across the reference genome, the mapping evenness was calculated (similar to evenness scores used to evaluate capture approaches as in [42,43,44]). The mapping evenness was calculated as the ratio between coverage (mapped sequence length divided by the size of the reference genome) and mean read depth (inferred from samtools depth). A high ratio indicates a relatively uniform mapping of reads to the reference genome, whereas a low ratio indicates that some positions of the reference genome are less well covered than others. To evaluate damage patterns of mapped reads, mapDamage (v2.0.7, [45]) was used. 

Read alignments were visualised, curated and consensus sequences were created using Geneious v.10.2.3 (https://www.geneious.com/). Consensus sequences were created based on the read alignment to the canid reference to which the highest number of reads could be mapped. A minimum read depth of three and a 50% consensus threshold were applied.

A multiple sequence alignment (MSA) was created by aligning the consensus sequences of the modern and ancient dhole samples to 41 canid sequences (Table S3, excluding the sequence of the Ethiopian wolf (*Canis simensis*) due to its high amount of missing data; [22,46,47,48,49,50,51,52,53]) using the MUSCLE algorithm [54] with default settings, as implemented in Geneious (v.10.2.3; https://www.geneious.com/). The MSA was modified as follows: removal of the control region based on the annotation of published genomes (Geneious) and removal of all columns containing gaps or uncalled positions (Ns) using MEGA (v5/v7; [55,56]). Thus, we restricted the analysis to positions covered by all samples. The MSA was used as an input for all following analyses. A maximum likelihood (ML) tree was built using the PHYML [57] (ML) plugin offered in Geneious (v.10.2.3; https://www.geneious.com/). The ML tree was reconstructed using a TIM2 + I + G model (specifically 010232), as it was identified as the best model using the Bayesian information criterion (BIC) by jModeltest (v2.1.7, [58,59]), using four γ categories, estimating the proportion of invariable sites and the γ distribution parameter, and optimising for topology, length and rate. A consensus tree was calculated using the implemented consensus tree builder in Geneious (v.10.2.3; https://www.geneious.com/) with default settings, applying a support threshold of 50%. Additionally, a Bayesian phylogeny was calculated using the MrBayes v3.2.6 [60] plugin offered in Geneious (v.10.2.3; https://www.geneious.com/) with the following settings: HKY model with four γ categories (best fitting model among the models offered by MrBayes, according to jModeltest [58,59] using the BIC), chain length of 11,000,000 with a subsampling frequency of 10,000. The results were inspected in Geneious checking for convergence after a burn-in phase of 1,100,000 and sufficient effective sample size (>200) for each parameter. A consensus tree was created using the consensus tree builder implemented in Geneious with a support threshold of 50% and a burn-in of 10%. In all phylogenetic reconstructions, the Gray fox (*Urocyon cinereoargenteus*) sequence served as outgroup.

To evaluate genetic diversity within species, pairwise differences were calculated for species for which four or more sequences were available (similar to [61]). Calculations were conducted in MEGA (v7; [56]), using default settings. Sequences were extracted from the aforementioned MSA. This was done for the coyote, African wild dog, gray wolf, and dhole. If ancient and modern sequences were available, the analysis was done separately for ancient and modern sequences, as well as once for all sequences together.

Sequences from samples Y-38 and Y-39 were compared to a 246 bp long dhole mitochondrial sequence fragment [21] by creating a media-joining haplotype network [62] with Popart [63]. In total, 25 sequences (Table S3) were used: 19 sequences from [21], the two ancient sequences and the one modern sequence from this study and the corresponding sequences from three dhole mitochondrial genomes available from Genbank. The MSA was created as described above.

## 3. Results

Sequencing after in-solution capture yielded between 1 and 5.5 million raw reads per sample. After pre-processing, between 997,439 and 4,199,293 reads were available for mapping. For the modern sample, about six million raw read pairs were generated, of which 5,242,575 could be merged and were used for mapping (Table S4).

BLAST results of pre-processed reads (prior to mapping) revealed a pattern typical for ancient samples (Figure S1). All ancient samples showed variable amounts of hits assigned to potential sources of environmental or post-excavation contamination, e.g., bacteria, cetartiodactyla, human, and fungi. In contrast, for the modern dhole and the modern wolf samples, substantially fewer hits were assigned to the aforementioned categories. When focussing on hits to canid lineages, for samples C3-6-2, C3-6-3, C3-6-4, and the modern gray wolf data from the SRA, the majority of hits were assigned to the gray wolf (Figure S1). For samples Y-37, Y-38, and Y-39, the majority of hits were assigned to environmental or post-excavation contaminant sources. Considering only hits assigned to canid lineages, samples Y-38 and Y-39 had more hits assigned to the dhole than to the gray wolf, whereas for sample Y-37, canid hits were solely assigned to the gray wolf (Figure S1). For the modern dhole sample, the majority of assigned hits were assigned to the dhole.

**Figure 1:**
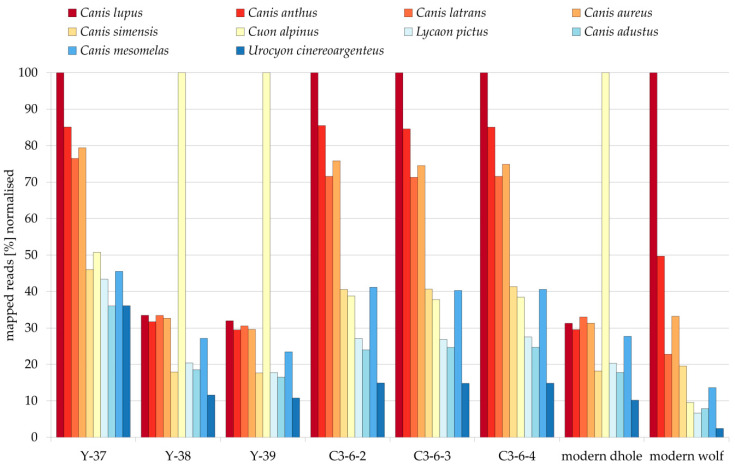
Percentage of reads mapping to various canid mitochondrial genomes for six ancient potential dhole samples, one modern dhole, and one modern gray wolf sample. Percentages were normalized to the highest number of mapping reads that was achieved for a canid mitochondrial genome (bars reaching 100%).

For the ancient samples, between 741 and 26,169 reads (median: 3894) could be mapped to various canid mitochondrial genomes (Figure 1, Table S5). For all but one sample, the highest number of reads mapping to mitochondrial genomes was achieved when using either the gray wolf or the dhole mitochondrial genome as a reference for mapping. Only for sample Y-37 a higher number of reads could be mapped to the human mitochondrial genome than to any canid mitochondrial genome (Figure S2). For this sample, the number of reads mapping to the human mitochondrial genome is comparable with other samples (e.g., Y-38). At the same time, the overall number of reads available for mapping (both the number of raw reads and the number of reads after pre-processing) is notably smaller (at least two times) in comparison to the other ancient samples (Figure 1, Table S5). It seems that sample Y-37 yielded only a low amount of (endogenous) DNA and exhibits a relatively high amount of contamination with human mitochondrial DNA. The canid mitochondrial genome to which the largest number of reads could be mapped for sample Y-37 was the gray wolf mitochondrial genome (Figure 1, Table S5). For Y-38 and Y-39, the highest number of reads could be mapped to the dhole mitochondrial genome, whereas for C3-6-2, C3-6-3, and C3-6-4 the highest numbers of reads were also mapped to the gray wolf mitochondrial genome (Figure 1, Table S5). All fossil samples showed slightly elevated substitution levels at read ends (Figures S3 and S4), which represent typical damage patterns for ancient samples [64,65]. The use of UDG/Endonuclease VIII during the library preparation has probably reduced the damage patterns [31]. 

To further describe the overall quality of the read alignments (visual representations in IGV v2.3.68, [66]: Figures S5 and S6), the mapping evenness was calculated. For the ancient samples, mapping evenness varied across all samples and all references between 0.18 and 0.99 (Table S5). Two samples, Y-38 and Y-39, reached the highest evenness value when mapped to the dhole mitochondrial genome (0.98 and 0.99, respectively). All other ancient samples showed the highest evenness value when reads were mapped to the gray wolf mitochondrial genome (Y-37: 0.93, C3-6-2: 0.99, C3-6-4: 0.98, C3-6-4: 0.99). 

**Figure 2:**
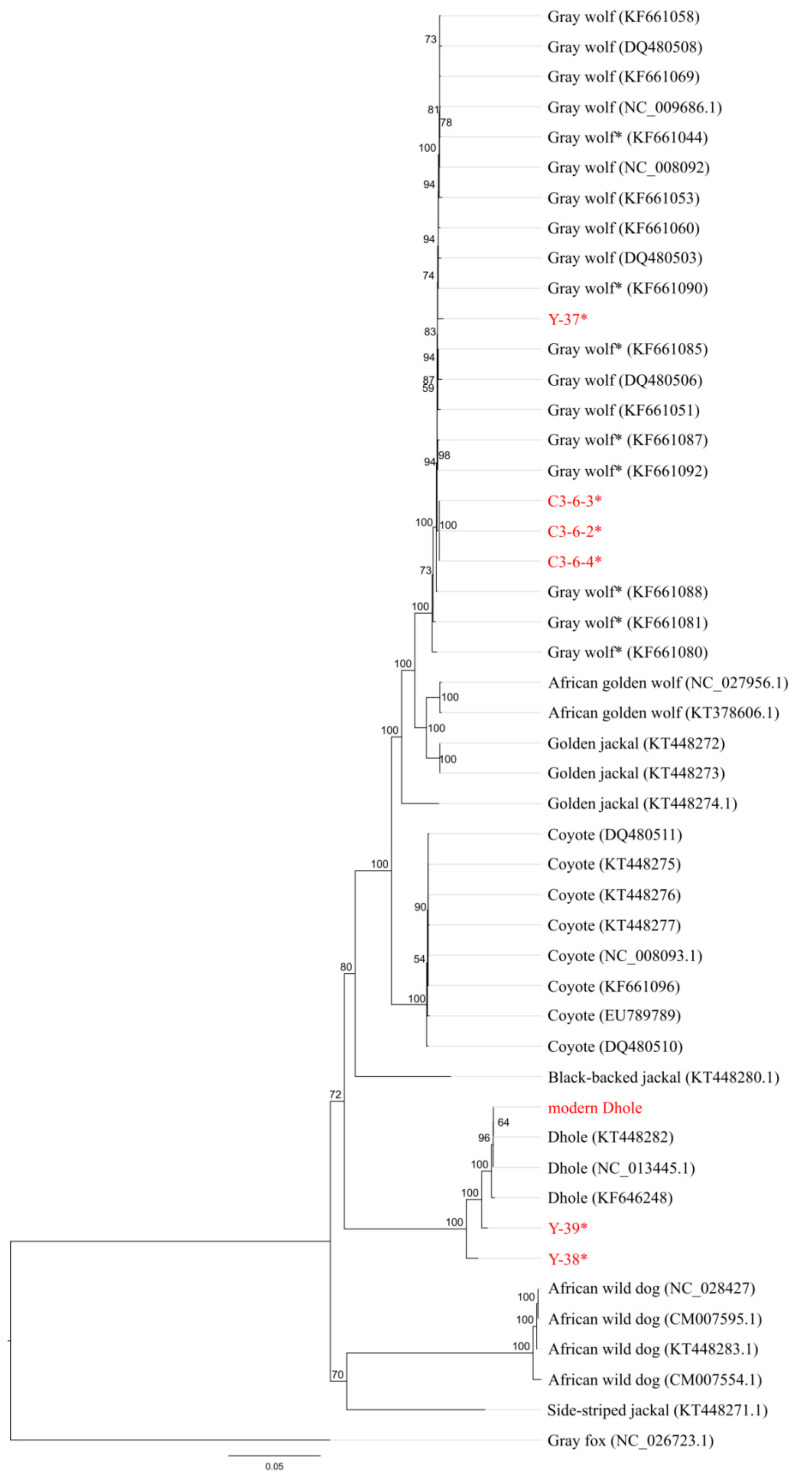
Maximum likelihood tree reconstructed with PHYML [57] using a 10,582 bp multiple sequence alignment (MSA) of 48 canid mitochondrial genomes as input and the TIM2 + I + G model with 100 bootstrap replicates. Support values are given next to nodes. Asterisks: ancient mitochondrial sequences. Red: samples of this study.

To reconstruct phylogenetic relationships between our samples and publicly available canid mitochondrial genomes (Table S3), ML (Figure 2) and Bayesian trees (Figure S7) were reconstructed based on an MSA of 10,582 bp length containing 48 sequences in total. Both trees show a quite similar topology and major canid clades can clearly be distinguished. The African wild dog clade and the side-striped jackal sequence are joined into one clade, which is the sister clade to a *Canis*-*Cuon* clade. This *Canis*-*Cuon* clade contains all other *Canis* and *Cuon* sequences used as well as all our samples. The published dhole sequences form the sister clade to a wolf–jackal–coyote clade, in line with previous results (e.g., [16,17]). Four of our six samples fall within the gray wolf clade, of which C3-6-2, C3-6-3, and C3-6-4 form a clade on their own, whereas Y-37 does not cluster with any specific gray wolf sequence. Only two ancient samples, Y-38 and Y-39, and the modern dhole sample, cluster with the published dhole sequences. Y-38 and Y-39 do not cluster together, but form two basal branches relative to the modern dhole sequences. The modern dhole sample is nested within the three publicly available dhole sequences. 

**Figure 3:**
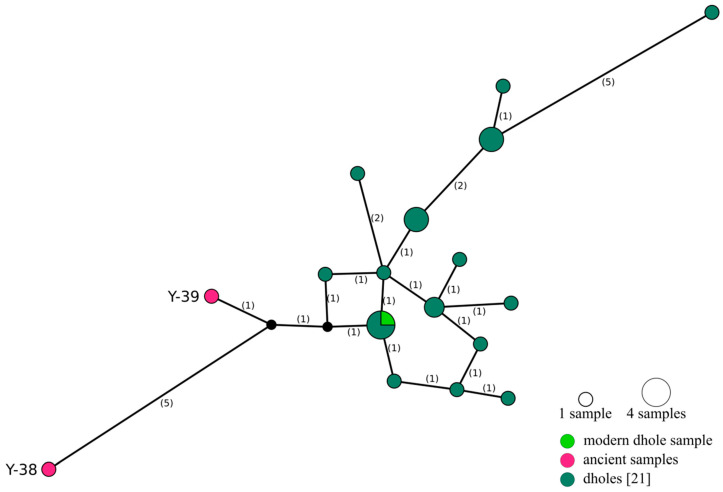
Median-joining network of a 170 bp long mitochondrial fragment of 25 dhole sequences. Number of mutations given in brackets. Red: ancient samples, light green: modern dhole sample, dark green: published dhole sequences [21], black: inferred (unsampled) haplotype.

To further investigate the relationship between samples Y-38 and Y-39 and modern dholes, we reconstructed a median-joining network using publicly available sequences of a 246 bp mitochondrial fragment (Figure 3). After the removal of uncalled positions and gaps from the MSA, only 170 bp remained, which contained 22 variable and eight parsimony-informative sites (sites containing at least two states that occur in at least two sequences each). The modern dhole sample is the same as the one from which Genbank sequence # AY682716 was obtained previously [21]. Unsurprisingly, both sequences are identical and they are also identical to two other sequences (KT448282, NC_013445), whereas Y-38 and Y-39 yielded unique and relatively diverged haplotypes. 

**Figure 4:**
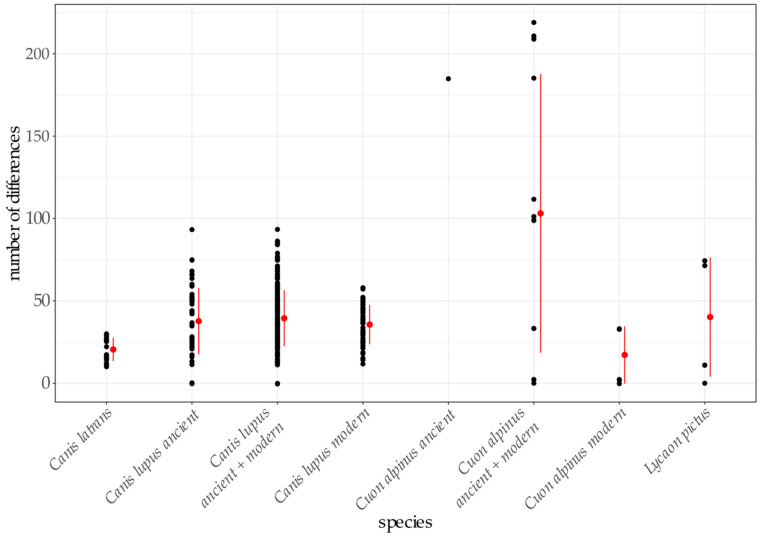
Pairwise differences as absolute number of differences between sequences for eight coyote, twelve ancient and ten modern gray wolf, two ancient and four modern dhole, and four African wild dog sequences are shown using MSAs of 10,582 bp length. Each black dot represents one pairwise comparison. Red dots are mean values and red lines indicate standard deviation.

To estimate the genetic diversity within and compare it between species, pairwise distances were calculated (Figure 4, Tables S6–S13). Based on the results of the phylogenetic analysis, samples Y-37, C3-6-2, C3-6-3, and C3-6-4 were incorporated into the set of ancient gray wolf sequences, while the samples Y-38 and Y-39 represented the set of ancient dhole sequences. The lowest amount of pairwise differences between sequences was measured for modern dhole sequences, which show only 17 differences on average (Table S7). The ancient dhole sequences had considerably more pairwise differences amongst them, more than ten times higher than amongst modern sequences (185 to 17; Tables S7–S9).

## 4. Discussion

### 4.1. Palaeogenetic Identification of Fossils 

The correct species identification of a fossil specimen can be of exceptional importance. Several studies have shown that one single sample can make a huge difference, e.g., the molecular confirmation of the first Late Pleistocene Eurasian *Homotherium* fossil [67], the identification of a Denisovan specimen as a further genetically distinct hominin lineage [68] or the identification of *Equus ovidovi* specimens outside the formerly known spatial and temporal range [61]. These findings enabled and called for a re-evaluation of the phylogenetic and phylogeographic history of the investigated (and closely) related species. 

Fossil specimens can be identified by morphological characters and measurements, by using molecular data or by a combination of both approaches (summarised and compared in [69]). Identification based on morphology is independent of the age of the specimen but demands a high level of expertise, is time-consuming, and can be heavily impeded by high fragmentation and incompleteness of specimens. Previous studies have shown that molecular data can provide valuable additional information to overcome the limitations of morphological species identification. For instance, in cases of highly fragmented or incomplete material, as for the Denisovan sample, phylogenetic relationships could be determined using mitochondrial data [68]. Samples that are too small and too numerous to be investigated morphologically can be processed as bulk samples and information can be gained even down to species level (e.g., [69,70]). However, the usability of molecular data heavily relies on DNA preservation, which depends both on the age of the sample and preservation conditions, among other factors. If possible, the most promising approach seems to combine both methods to maximise the available information for a specimen (e.g., [69,71]).

In all ancient samples, DNA from exogenous and contaminant sources was present (Figure S1), which is common for ancient DNA samples (e.g., [33,72]). Sources of exogenous DNA are, e.g., post-mortem microbial colonisation and the transfer of modern human DNA during sample handling. The degree of contamination can be highly variable (e.g., [33,72]) and depends on multiple factors, among others: conditions at the finding site, handling during excavation and examinations, and storage conditions. Thus, it is not surprising that the ancient samples show different levels of exogenous DNA. These various types of contamination need to be taken into account during data processing. However, the read alignment tool used (BWA aln) has been shown to be quite robust against the mapping of contaminant reads to the reference sequences of the source material [73].

We used three different lines of evidence to evaluate the species affiliation of our samples: (1) the amount of reads that could be mapped to different canid reference mitogenomes (Figure 1, Table S5), (2) the mapping evenness along the reference (Figures S5 and S6, Table S5), and (3) the position of samples in phylogenetic trees (Figure 2, Figure S7). All three approaches confirm that of the six putative dhole samples, four are actually fossils of gray wolves and only two are indeed dholes. Both the number of reads that could be mapped to and the evenness of the coverage of a reference give an indication how well reads are fitting to a reference, i.e., how similar reads and references are. Both approaches rely on the availability of reference sequences, as the species the fossil belongs to has to be included in the comparison. This is nicely shown by the fact that the ancient and modern dhole samples show similarly high numbers of reads mapped to four of the *Canis* species (*Canis lupus*, *Canis anthus*, *Canis latrans*, and *Canis aureus*; Figure 1, Table S5) in the comparison, but much higher values for the dhole reference mitogenome (at least three times more). The effect is much weaker, but still present for the ancient wolf samples (C3-6-2, C3-6-3, C3-6-4, Y-37, Figure 1, Table S5), compared to the three most closely related *Canis* species. If the fossil species is not included in the references, this can give a misleading result, as the closest related species is likely to yield the highest number of mapping reads. Therefore, careful evaluation of the available reference species is required when trying to identify fossils using this approach. Canids are a well-sampled group, in which mitochondrial genomes are available for most of the currently described species, but this is not necessarily always the case, especially for the species-rich, small mammal groups like rodents or shrews.

The evenness of the read alignment is a second possibility to evaluate species identification. If sequence reads are mapped to an intraspecific mitogenome or very closely related species, even mapping of reads across the entire reference genome is expected, resulting in little variability in the level of coverage. Highly variable regions (e.g., control region) or repetitive elements would lead to lower or higher coverage, but these represent only a small portion of the mitochondrial genome. If the reference used for mapping is more distantly related to the sample, fewer reads will be mapped and only to conserved sites at which sequence similarity is high, leading to uneven coverage (compare Figures S5 and S6). Therefore, we evaluated evenness visually, and quantified it as the ratio of coverage against read depth. The latter is the more useful method when using larger reference sequences, e.g., exons or the whole genome, when visual inspection of the mapped reads becomes unwieldy or even impossible.

A potential drawback of the evenness-of-coverage metric exists for cases when cross-species hybridisation enrichment was applied in the laboratory to enrich for a particular genetic region (e.g., mitogenome, exome). It has been shown that cross-species capture—i.e., when baits from a related species are used for enrichment—can introduce strong biases for regions with the highest sequence similarity between bait and target [74,75]. We utilised hybridization capture with dhole bait DNA, which means that the four samples that were post-hoc identified as wolf were captured with a cross-species capture approach. However, the mitogenomes of dhole and wolf are highly similar (average sequence identity 92%), which has not led to a significant reduction in the coverage of lower-identity regions in the alignment (see Figures S5 and S6).

Finally, the phylogenetic analysis also supports the species affiliations of the ancient samples suggested by the other two approaches. In this context, it needs to be noted that many studies have revealed evidence for more or less extensive gene flow between different members of the genus *Canis* (e.g., [15,16,22,41,76]). Since the mitochondrial genome is inherited strictly maternally, hybridisation between two species can lead to contradictory signals on the mitochondrial and nuclear level, e.g., the occurrence of coyote mitochondrial genomes within a wolf population or vice versa (e.g., [77]). Consequently, in cases of hybridisation, analysing only the mitochondrial genome may lead to incorrect species identification and may also be an explanation for discrepancies between molecular and morphological species identification. If in doubt, the analysis of mitochondrial and nuclear markers enables a more precise analysis. However, to our knowledge, hybridisation events between dholes and wolves have not been reported to date. Although there is a considerable overlap in the distribution of dholes and wolves, little is known if and in case how both species interact [23]. However, previous studies on the nuclear level found evidence for interspecific admixture events with the involvement of the dhole. They describe potential ancient admixture between dhole and African wild dog despite the fact that there is, to date, no evidence that these two species ever overlapped in range [15]. The same study found evidence for an increased genetic affinity between the gray wolf-coyote-lineage and dhole, suggesting different scenarios of ancient gene flow between ancestors of these lineages. Another study found gene flow from an ancient lineage, maybe dhole, into a wolf from Southern China [78]. These findings underline both the importance and potential of nuclear data from fossil dhole specimens to elucidate not only the phylogenetic history of dholes but also of other canid species.

### 4.2. (Mis) Identification of Dhole Remains

Although our dataset is small, the result that only two out of six samples could be confirmed as dhole highlights the potential challenges involved with species identification based on morphological data alone. These challenges are also evident from the palaeontological literature, where re-identification of dhole fossils to wolf or other canid species, and vice versa, is not uncommon (e.g., [5,7,8,10]). The identification of dhole fossils, in particular of postcranial elements, is complicated by the overlap in size, and sometimes only subtle morphological differences between dholes and other canid species, including the gray wolf (e.g., [3,5,7,8,79]). Therefore, some studies have even limited their analysis to cranial bones and teeth due to the difficulty in identifying postcranial dhole remains [7]. 

Cranial elements are considered easier to identify, as the cranium, mandible and dental composition often contain characteristic features unique to a particular species (see Tables 1 and 7 in [8] for a summary of morphological characters of the cranium and mandibles). However, three of the six putative dhole samples were cranial elements, two mandibles and one skull (Table 1), and yet only two of these could be confirmed as dholes by the molecular analysis. The absence of the lower third molar is seen as a prominent feature needed to identify dhole fossils by some authors (e.g., [79]). However, this feature is not always available. Sample Y-37 was missing the posterior part of the mandible and was morphologically identified as dhole using other morphological characteristics (e.g., rather short jaw, crowded teeth; Roblíčková, personal observation). Moreover, previous studies have shown similarity and overlap in size for some morphological features of the cranium between dhole and wolf (e.g., [8]). In fact, the presence of relatively small and gracile wolf specimens was documented at the finding site of Y-37 (Marciszak, unpublished data). Both facts complicate an unambiguous identification by morphological means and can explain the contradictory results of the morphological and molecular species identification for this sample. For samples Y-38 and Y-39, both lacking the lower third molar, morphological and molecular species identification are in agreement. However, wolves without the lower third molar have occasionally been documented, too [80,81,82]. These studies either rely on complete skulls [80] or on material from extant wolf populations from Eastern Europe [81,82], so confusion with dhole seems highly unlikely. The loss of the third molar and the development of hypercarnivorous features was a complex evolutionary process. Descriptions of dhole fossils of different geological ages seem to underpin the gradual development of typical dhole features [3,10]. However, it is not always clear if (the condition of) the described material allows an unambiguous species identification. Nevertheless, previous studies have described morphological data from fossil dholes that indicate variability in the degree of hypercarnivory in different populations or lineages [7,79]. Both cited studies focus on cranial elements, comparing dhole fossils with other dhole fossils and fossils from extant and extinct canids. In these studies, the species identification of the described dhole fossils relies on the evaluation of a range of diagnostic characteristics (in addition to the missing third molar).

Postcranial elements are even more difficult to assign to species, although the degree of overlap in postcranial skeletal elements between different canid species varies for different skeletal elements. Three of our samples were postcranial elements (metapodia, Table 1), and all three were re-identified as originating from gray wolf. A recent study on the morphological variability in living and fossil canids showed that there is some overlap in the dimensions of some metapodial elements of dholes and wolves, with the wolf being larger than the dhole, while other elements differ considerably [5]. It should be noted, however, that this study included only two fossil dhole specimens and a single wolf sample. Therefore, it probably does not represent the full size range of the species [5] and almost certainly underestimates the size overlap of the two species. The metapodia included in our study fell within the lower range of the size range found in wolves, thus representing rather small-bodied wolves (Drăgușin and Vasile, personal observation).

Although our results should be considered preliminary due to the small sample size, they could suggest that a considerable number of putative dhole fossils actually belong to the gray wolf. If this were indeed the case, the dhole would be even rarer in the late Pleistocene fossil record (Table S14; [5,6,7,24,83,84,85,86,87,88,89]) than currently recognised. Alternatively, the challenges we highlight in distinguishing dhole and wolf fossils could also suggest that the low frequency or absence of dhole fossils from certain regions (e.g., in Poland or Romania; [5]) might be explained by the misidentification of dhole fossils as Canidae or *Canis* sp. (e.g., [5,8]). Due to the small sample size, our results do not allow to draw conclusions about the Pleistocene distribution of dholes. Thus, genetic analysis of additional putative dhole and other canid fossil specimens, especially of specimens with uncertain species affiliation, would be crucial to define the spatial and temporal occurrence of dhole across Europe more precisely. Moreover, secure identification of dhole fossils would also increase the number of specimens available for morphological measurements, completing and refining our knowledge of the morphological variability of Pleistocene dholes as well as increasing the body of genetic data available for comparative analysis. 

### 4.3. Relationship between Ancient and Modern Dholes

To our knowledge, the data gained from the two samples identified as dholes represent the first ancient DNA sequences published for this species. However, due to a lack of modern data available for comparison, only limited population genetic insights or phylogeographic comparisons are possible at present. To date, genetic data from dholes have been limited to a small number of modern and historical samples (e.g., [15,16,17,18,19,20,22]). Only one published study has investigated the phylogeographic structure of dholes, extracting DNA from non-invasive faecal samples of wild dholes and also including DNA from captive individuals and historical samples [21]. 

Phylogenetic analysis of complete mitochondrial genomes from the two ancient, one newly sequenced, and three published modern dholes reveals considerable differences between ancient and modern dhole sequences (Figure 4, Table S9) and places the ancient sequences at a basal position to the modern sequences in phylogenetic trees (Figure 2, Figure S7). Analysis of short mtDNA sequences allowed for the inclusion of more samples, and further confirms the divergence of the ancient samples (Figure 3). The network does not reveal the structure previously found in the mtDNA sequences [21] due to the loss of information through the reduction of the underlying alignment to only 170 bp. Thus, the results of this analysis need to be taken with some caution and do not allow insights into population structure. Nevertheless, the position at the edge of the network and the assumption of two inferred (unsampled) haplotypes separating the ancient from the modern dhole sequences are in line with their basal position in the phylogenetic trees obtained from much longer mitochondrial sequences (Figure 2 and Figure S7). Modern dholes show little genetic diversity, and show the lowest number of differences of all canid species analysed in this study (Figure 3 and Figure 4, Tables S6–S13). The sequences of the ancient dhole samples add a considerable amount of previously unknown genetic variability, suggesting that substantial genetic diversity was lost during the transition from Pleistocene to recent dhole populations. It has to be pointed out that our comparisons of pairwise distance have to be evaluated with caution, since the number of available sequences is low and not the entire distribution is covered. Thus, the diversity within species might not be reflected to its full extent. However, a loss of genetic diversity over time has been documented for other species [90,91,92], including wolves in North America [93] and Europe [94], leopards [2], giant pandas [95] and South American mammals [96]. Our findings indicate a certain genetic separation between Asian and European dholes. However, an in-depth analysis requires more genetic data from modern and ancient samples, covering a wide geographic range.

The approximate age of the two verified dhole specimen (35 to 45 ka, Table 1) predates the extinction of the dhole in Europe, which is assumed to have taken place at the end of the Late Pleistocene [3]. The presumably most recent European dhole remains have been found in Italy and were dated to 10,870 ± 119 B. P. ([7] and references therein). However, a reliable determination of their extinction time requires the dating of more reliably identified dhole specimens. We can only speculate on the reasons leading to the extinction of the dhole in Europe. The hypercarnivorous lifestyle of the dhole constitutes a highly adapted and specialised feeding behaviour. Previous studies have shown that hypercarnivorous wolf ecomorphs experienced either extinction (North America) or considerable reduction (Europe) at the end of the Pleistocene, probably strongly related to the extinction of large-bodied prey [93,94]. Extant dholes are known to prey on a large variety of prey from small rodents to gaur and sambar (e.g., [97,98]). How far this feeding behaviour relates to Pleistocene dholes is unclear and requires further investigation, e.g., focussing on faunal assemblages and stable isotope analysis. Finally, additional factors like habitat preferences may have contributed to the extinction of the dhole from most of its Pleistocene distribution.

## 5. Conclusions

In this study, we reconstructed mitochondrial genomes of six fossil specimens initially identified as dhole and found that more than half had to be reassigned as gray wolves. This underlines the importance of genetic analysis for the identification of fossil specimens, especially if findings originate from postcranial elements or are highly fragmented and species affiliation is difficult to determine. Phylogenetic analyses point towards a considerable divergence between ancient and modern dhole sequences and a loss of genetic diversity in modern dholes. However, the database of modern dhole sequences available for comparative analysis is scarce. The generation of further genetic data, both mitochondrial and nuclear, on the population level, is mandatory to evaluate the findings of the current study and extend our knowledge of the phylogenetic history and genetic diversity of the dhole. 

## Figures and Tables

**Table 1:** Sample details for six ancient putative and one modern dhole sample. Additional sample information can be found in Table S1.

Sample	Element	Location	Approx. Age	Reference
Y-37	mandible	Jáchymka cave, Czech Republic	ca. 35–45 ka (co-occurring remains have been dated, Marciszak, unpublished data)	[25]
Y-38	skull	Jáchymka cave, Czech Republic	ca. 35–45 ka (co-occurring remains have been dated, Marciszak, unpublished data)	[25]
Y-39	mandible	Bacho Kiro Cave, Bulgaria	ca. 39–45 ka (multiple samples from the same layer have been dated, e.g., [28])	[26]
C3-6-2	metapodial	Peștera Seacă din Ogașul Stoienilor, Romania	ca. 25 ka (a mandible found close by was dated)	[27]
C3-6-3	metapodial	Peștera Seacă din Ogașul Stoienilor, Romania	ca. 25 ka (a mandible found close by was dated)	[27]
C3-6-4	metapodial	Peștera Seacă din Ogașul Stoienilor, Romania	ca. 25 ka (a mandible found close by was dated)	[27]
modern dhole	blood (provided as DNA extract)	Allwetterzoo Münster	Recent	[21]

## Data Availability

Sequencing data has been made available on the NCBI Sequence read archive (SRA) under the BioProject accession number PRJNA691169.

## Supplementary Materials

The following are available online at https://www.mdpi.com/2073-4425/12/2/144/s1, Figure S1: Blast results for pre-processed reads (prior to mapping) of six ancient putative dhole samples, one modern dhole and one modern gray wolf sample. Lineages to which at least one percent of hits were assigned at least for one of the samples are shown. Lineages with fewer assigned reads are not included, Figure S2: Percentage of reads mapping to various canid mitochondrial genomes and one human mitochondrial genome for six ancient potential dhole samples, one modern dhole, and one modern gray wolf sample. Percentages were normalized to the highest number of mapping reads that was achieved to a canid mitochondrial genome (bars reaching 100%), Figure S3: Damage patterns inferred with mapDamage (v2.0.7; [45]) for samples C3-6-2, C3-6-3, C3-6-4 and Y-37. All samples mapped against a gray wolf mitochondrial genome (NC_009686.1). Red: C to T substitutions, blue: G to A substitutions, grey: other substitutions. All samples show slightly elevated substitution levels at read ends (i.e., C to T and G to A substitutions), Figure S4: Damage patterns inferred with mapDamage (v2.0.7, [45]) for samples Y-38 and Y-39. Samples Y-38 and Y-39 mapped against a dhole mitochondrial genome (NC_013445.1). Red: C to T substitutions, blue: G to A substitutions, grey: other substitutions. Y-38 and Y-39 show slightly elevated substitution levels at read ends (i.e., C to T and G to A substitutions), Figure S5: Coverage of the wolf mitochondrial reference genome when mapping reads of six ancient samples, one modern dhole and one modern wolf sample. Taken from the “Coverage track” in IGV (v 2. 3.68; [66]). Y axes are differing across panels (note minimum and maximum read depth given in the left upper corner of each coverage track). Coloured lines indicate variable positions (i.e., differences between reference and reads aligned), Figure S6: Coverage of the dhole mitochondrial reference genome when mapping reads of six ancient samples, one modern dhole and one modern wolf sample. Taken from the “Coverage track” in IGV (v2.3.68; [66]). Y axes are differing across panels (note minimum and maximum read depth given in the left upper corner of each coverage track). Coloured lines indicate variable positions (i.e., differences between reference and reads aligned), Figure S7: Bayesian tree using an MSA of 48 sequences and 10,582 bp length as input, applying a chain length of 11,000,000 with a subsampling frequency of 10,000. The consensus tree was created using a support threshold of 50% and a burn-in of 10%. Posterior probabilities are given at nodes. Red: samples. Asterisk: ancient sequences, Table S1: Additional sample details for six ancient and one modern sample, Table S2: Sequences and characteristics of primers used for LR PCRs. Primers were designed using the web interface of Primer3Plus [29]. length = length of primer in base pairs (bp); Tm = melting temperature in °C; GC = percentage of the bases C and G, Table S3: Mitochondrial genomes and control region sequences used to create MSAs and to reconstruct phylogenies and networks. If not indicated otherwise sequences were only used to reconstruct phylogenies. Bold printed genomes were used as references for read mapping, Table S4: Pre-processing of raw reads including adapter trimming with cutadapt (v1.12; [36]) and merging of paired end reads with flash (v1.2.10; [37]), Table S5: Mapping results of eight samples using BWA aln (v0.7.8; [39]) with default settings and various canid mitochondrial genomes and the human mitochondrial genome as references for mapping, Table S6: Estimates of evolutionary divergence between eight modern coyote sequences. The absolute number of differences between any pair of sequences is shown. All positions containing gaps and missing data were eliminated. There was a total of 10,582 positions in the final dataset. Additionally, the minimum, average and maximum number of differences are given. Analyses were conducted in MEGA (v7; [56]), Table S7: Estimates of evolutionary divergence between four modern dhole sequences, Table S8: Estimates of evolutionary divergence between two ancient dhole sequences, Table S9: Estimates of evolutionary divergence between four modern and two ancient dhole sequences, Table S10: Estimates of evolutionary divergence between ten modern gray wolf sequences, Table S11: Estimates of evolutionary divergence between eight ancient gray wolf sequences and the sequences of four ancient samples, Table S12: Estimates of evolutionary divergence between ten modern and twelve ancient gray wolf sequences, Table S13: Estimates of evolutionary divergence between four modern African wild dog sequences, Table S14: List of known dhole fossil sites. This list is not intended to be exhaustive and mainly focussed on publications which are summarising dhole fossil sites in Eurasia and North America.
