# Supplementary material for: Ancient DNA from the Asiatic Wild Dog (Cuon alpinus) from Europe
Source: Genes (Basel). 2021 Jan 22;12(2):144. doi: 10.3390/genes12020144 (PMC7911384; doi:10.3390/genes12020144)
Supplement: Supplementary file 1 [file genes-12-00144-s001.zip › Ancient DNA from the Asiatic wild dog Cuon alpinus from Europe_Manuscript_Supplements_proof_21.01.2021.pdf]

genes

MDPI

Supplementary Materials for:

Ancient DNA from the Asiatic wild dog (*Cuon alpinus*) from Europe

Ulrike H Taron, Johanna L A Paijmans, Axel Barlow, Michaela Preick, Arati Iyengar, Virgil Drăgușin, Ștefan Vasile, Adrian Marciszak, Martina Roblíčková and Michael Hofreiter

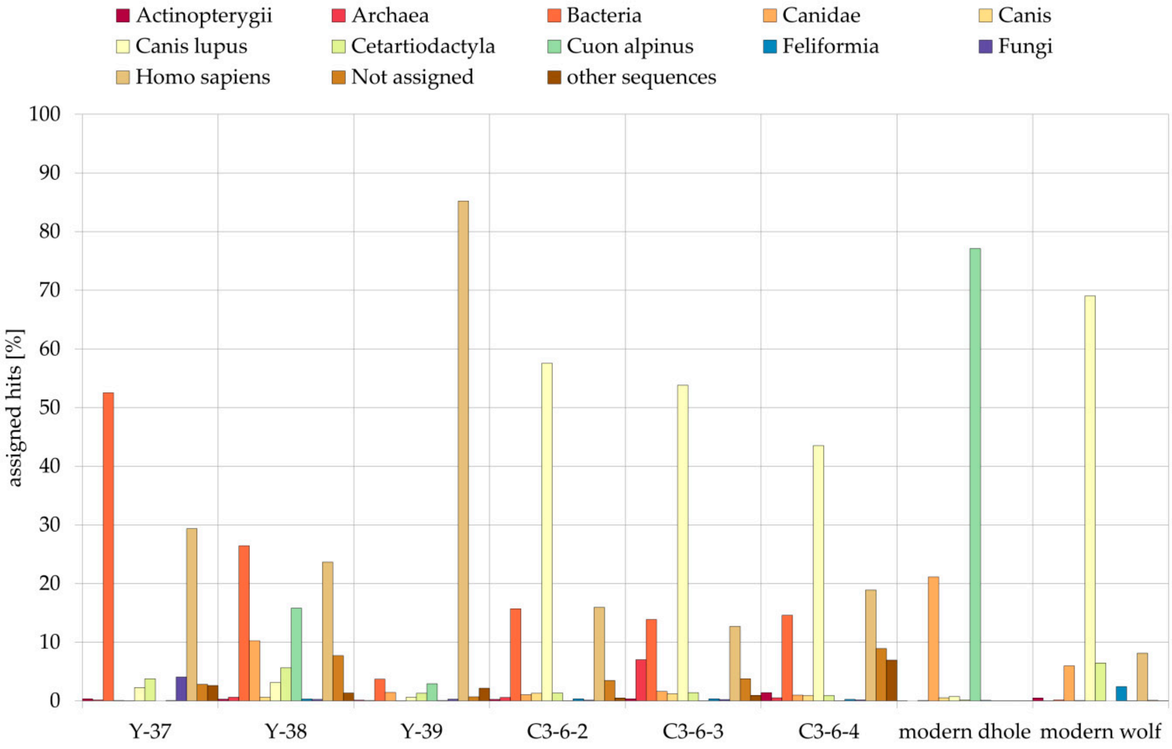

Figure S1. Blast results for pre-processed reads (prior to mapping) of six ancient putative dhole samples, one modern dhole and one modern gray wolf sample. Shown are lineages to which at least one percent of hits were assigned at least for one of the samples. Lineages with less assigned reads are not included.

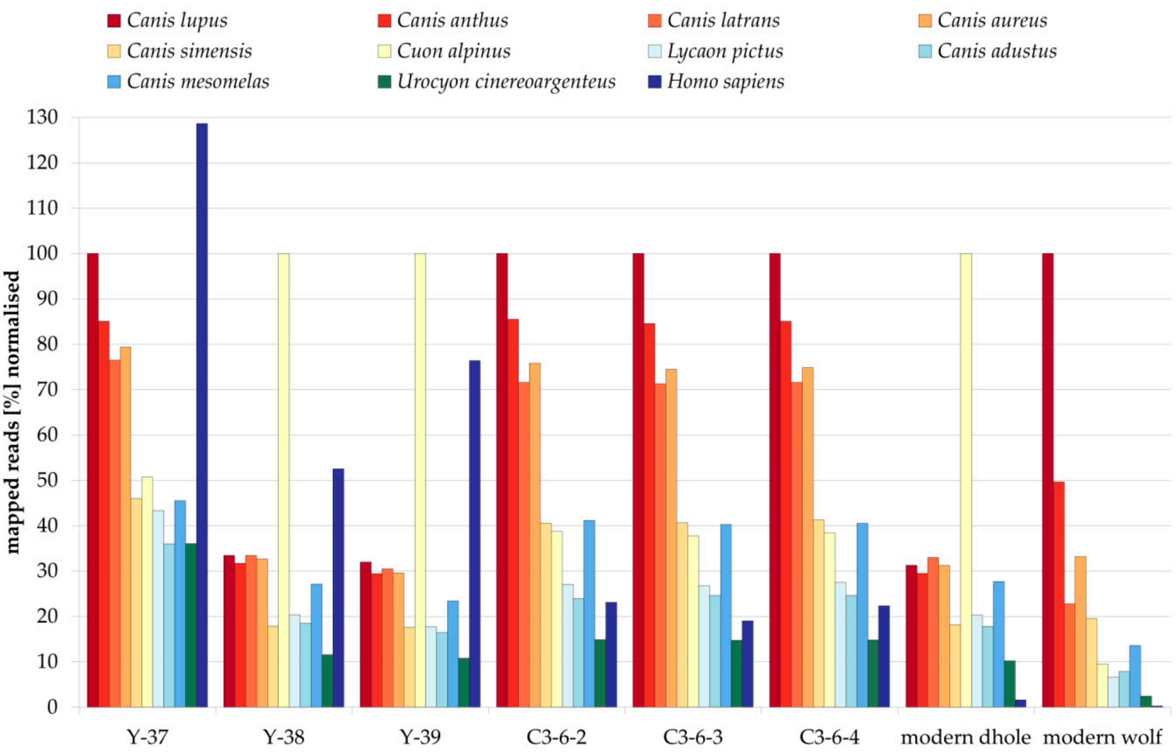

Figure S2. Percentage of reads mapping to various canid mitochondrial genomes and one human mitochondrial genome for six ancient potential dhole samples, one modern dhole, and one modern gray wolf sample. Percentages were normalized to the highest number of mapping reads that was achieved for a canid mitochondrial genome (bars reaching 100 %).

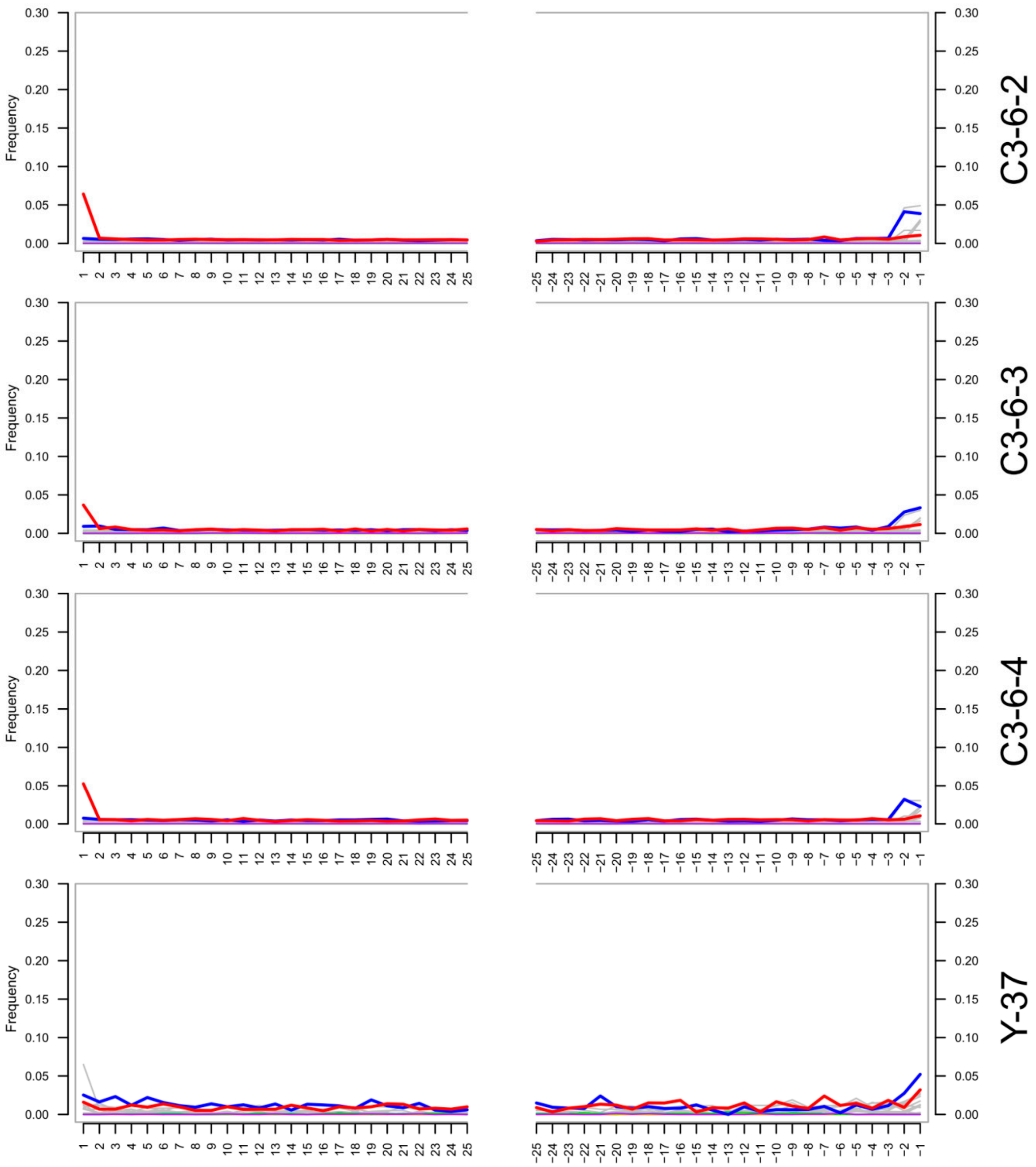

Figure S3. Damage patterns inferred with mapDamage (v2.0.7; [45]) for samples C3-6-2, C3-6-3, C3-6-4 and Y-37. All samples mapped against a gray wolf mitochondrial genome (NC_009686.1). Red: C to T substitutions, blue: G to A substitutions, grey: other substitutions. All samples show slightly elevated substitutions levels at read ends (i.e. C to T and G to A substitutions).

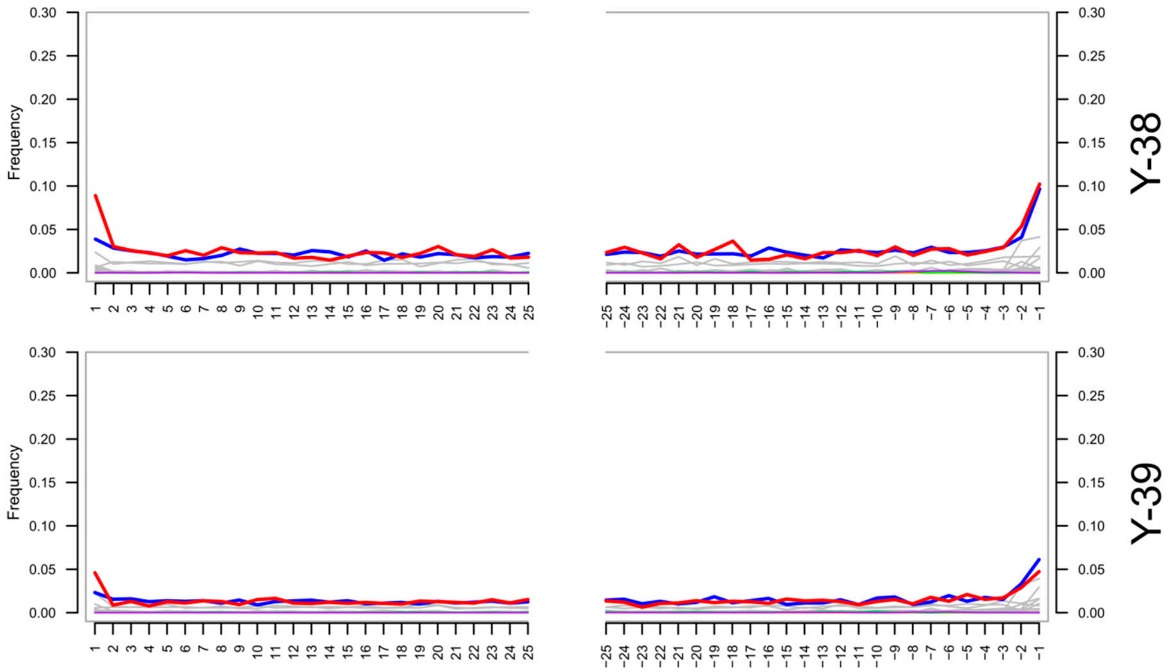

Figure S4. Damage patterns inferred with mapDamage (v2.0.7, [45]) for samples Y-38 and Y-39. Samples mapped against a dhole mitochondrial genome (NC_013445.1). Red: C to T substitutions, blue: G to A substitutions, grey: other substitutions. Y-38 and Y-39 show slightly elevated substitutions levels at read ends (i.e. C to T and G to A substitutions).

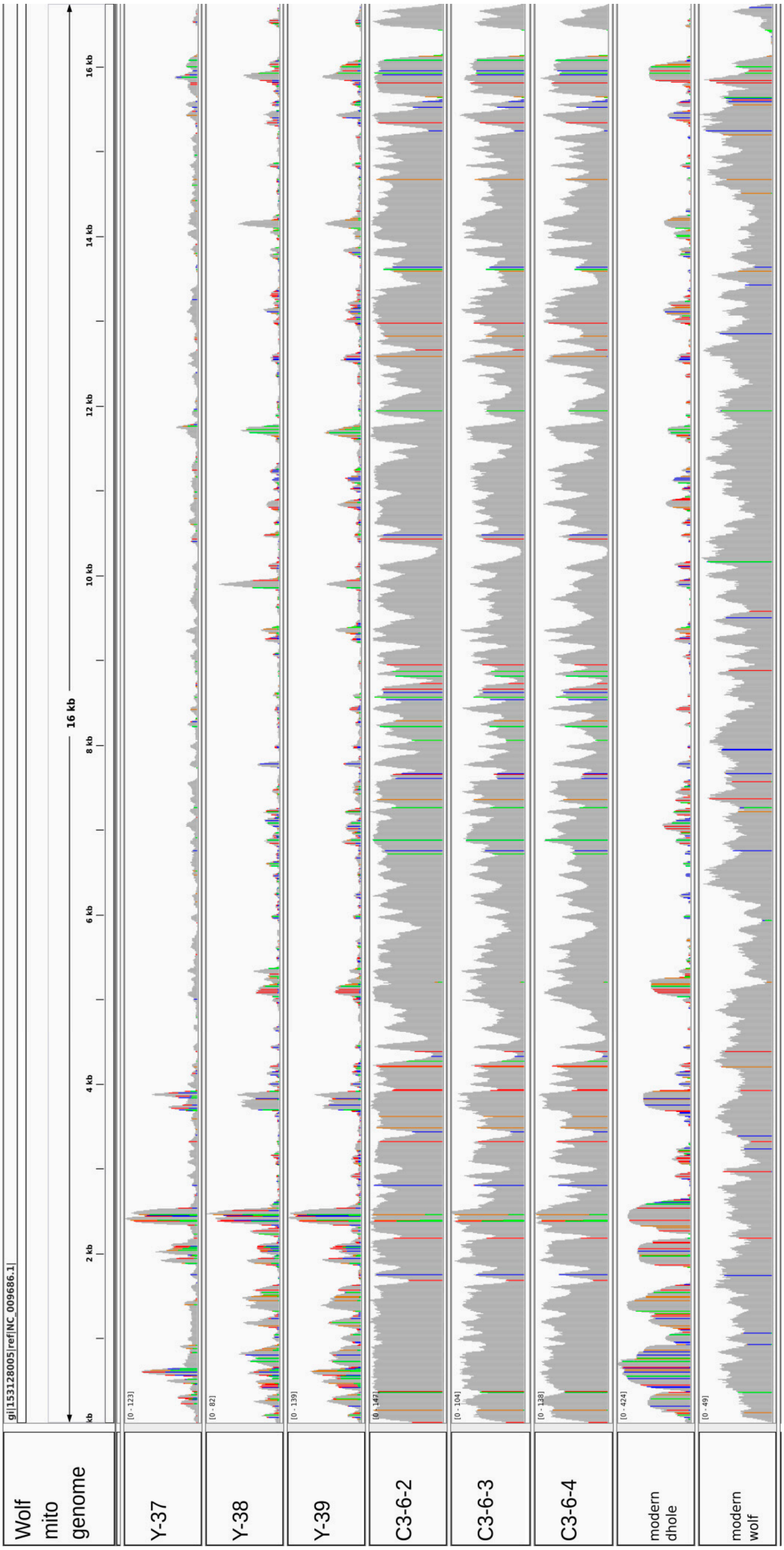
Wolf mito genome
gi|153128005|ref|NC_009686.1|
16 kb
2 kb
4 kb
6 kb
8 kb
10 kb
12 kb
14 kb
16 kb
Y-37
Y-38
Y-39
C3-6-2
C3-6-3
C3-6-4
modern dhole
modern wolf

Figure S5. Coverage of the wolf mitochondrial reference genome when mapping reads of six ancient samples, one modern dhole and one modern wolf sample. Taken from the "Coverage track" in IGV (v2.3.68; [66]). Y axes are differing across panels (note minimum and maximum read depth given in the left upper corner of each coverage track). Coloured lines indicate variable positions (i.e. differences between reference and reads aligned).

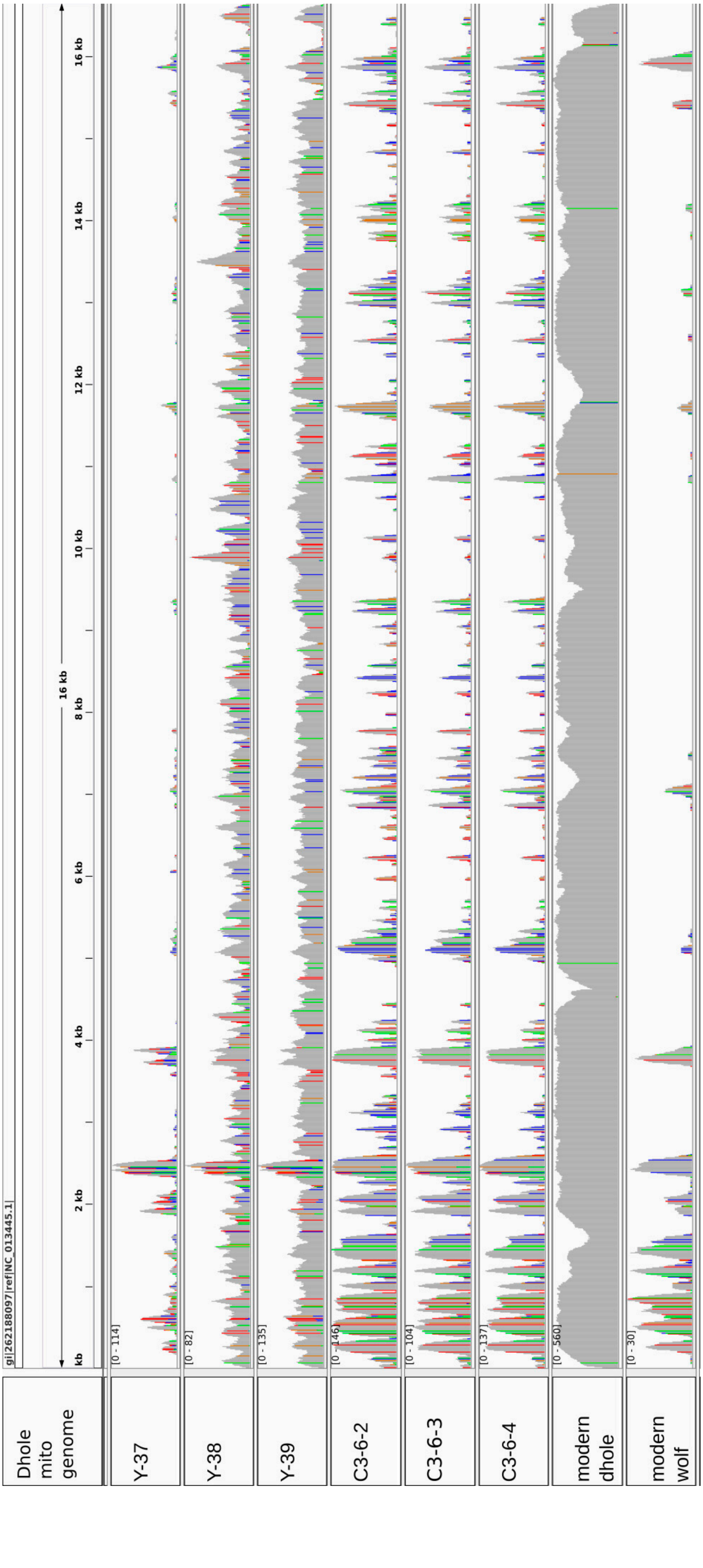
Dhole mito genome
Y-37
Y-38
Y-39
C3-6-2
C3-6-3
C3-6-4
modern dhole
modern wolf
gi|262188097|ref|NC_013445.1|
16 kb
kb
2 kb
4 kb
6 kb
8 kb
10 kb
12 kb
14 kb
16 kb
[0 - 114]
[0 - 82]
[0 - 135]
[0 - 146]
[0 - 104]
[0 - 137]
[0 - 560]
[0 - 30]

Figure S6. Coverage of the dhole mitochondrial reference genome when mapping reads of six ancient samples, one modern dhole and one modern wolf sample. Taken from the "Coverage track" in IGV (v2.3.68; [66]). Y axes are differing across panels (note minimum and maximum read depth given in the left upper corner of each coverage track). Coloured lines indicate variable positions (i.e. differences between reference and reads aligned).

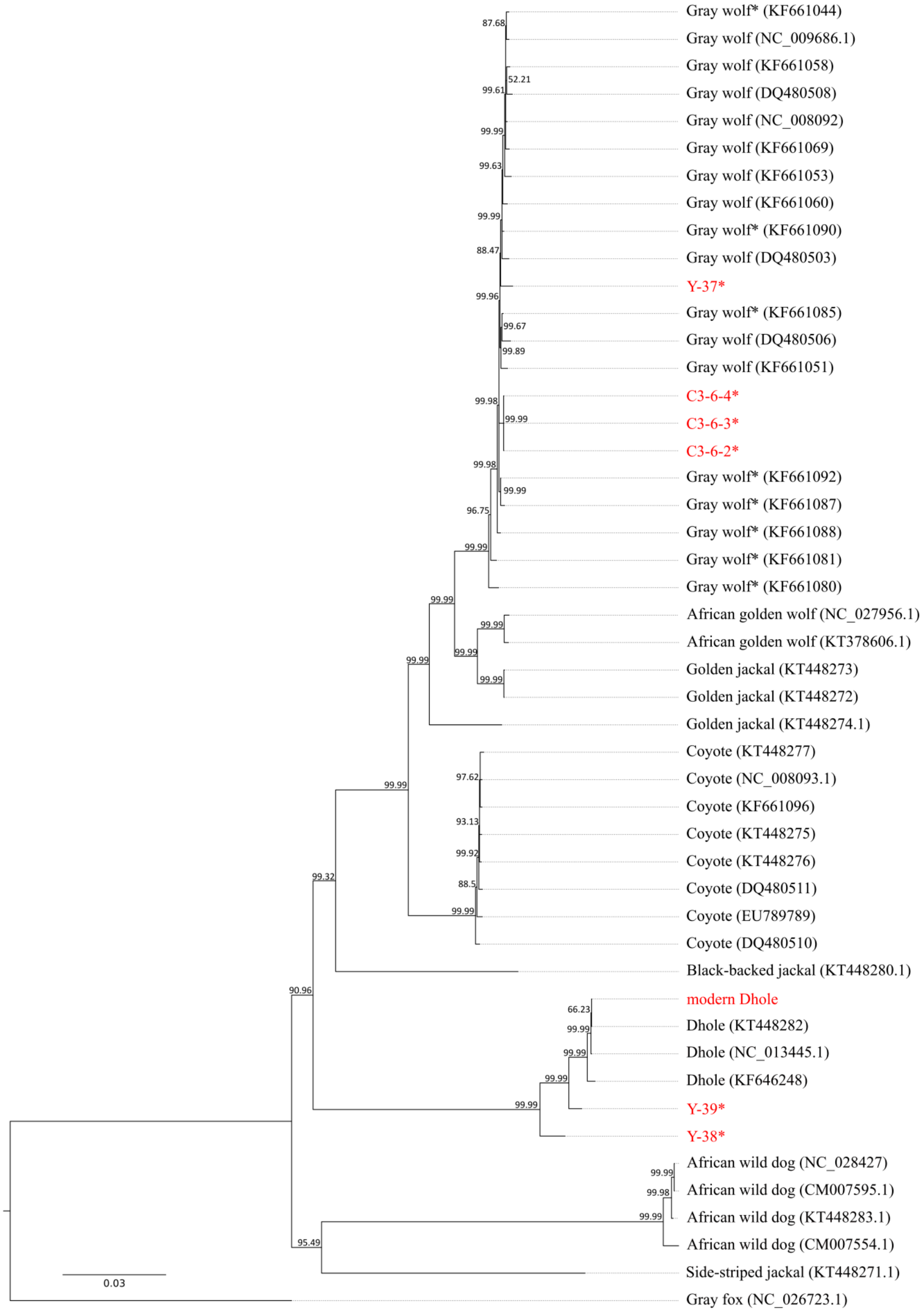

Figure S7. Bayesian tree using an MSA of 48 sequences and 10,582 bp length as input, applying a chain length of 11,000,000 with a subsampling frequency of 10,000. The consensus tree was created using a support threshold of 50% and a burn-in of 10%. Posterior probabilities are given at nodes. Red: samples. Asterisk: ancient sequences

References (as numbered and listed in the main text)

5. Ghezzo, E.; Rook, L. *Cuon alpinus* (Pallas, 1811) (Mammalia, Carnivora) from Equi (Late Pleistocene, Massa-Carrara, Italy): Anatomical Analysis and Palaeoethological Contextualisation. *Rend. Fis. Acc. Lincei* **2014**, *25*, 491–504, doi:10.1007/s12210-014-0345-6.
7. Petrucci, M.; Romiti, S.; Sardella, R. The Middle-Late Pleistocene *Cuon* Hodgson, 1838 (Carnivora, Canidae) from Italy. *Bollettino della Societa Paleontologica Italiana* **2012**, *51*, 137–148, doi:10.4435/BSPI.2012.15.
21. Iyengar, A.; Babu, V.N.; Hedges, S.; Venkataraman, A.B.; Maclean, N.; Morin, P.A. Phylogeography, Genetic Structure, and Diversity in the Dhole (*Cuon alpinus*). *Molecular Ecology* **2005**, *14*, 2281–2297, doi:10.1111/j.1365-294X.2005.02582.x.
22. Koepfli, K.-P.; Pollinger, J.; Godinho, R.; Robinson, J.; Lea, A.; Hendricks, S.; Schweizer, R.M.; Thalmann, O.; Silva, P.; Fan, Z.; et al. Genome-Wide Evidence Reveals That African and Eurasian Golden Jackals Are Distinct Species. *Current Biology* **2015**, *25*, 2158–2165, doi:10.1016/j.cub.2015.06.060.
25. Stehlík, A. *Cuon eauropaeus* Bourguignat z Plistocenních Usazenin Jeskyně Jáchymky v Josefském Údolí u Adamova. *Práce Moravské Přírodověckě Společnosti* **1944**, *16*, 1–21.
26. Wiszniowska, T. Carnivora. In *Excavation in the Bacho Kiro Cave (Bulgaria). Final report.*; Państwowe Wydawnictwo Naukowe: Warszawa, 1982; pp. 52–55.
27. Drăgușin, V.; Vasile, Ștefan; Terhune, C.; Petculescu, A.; Robu, M.; Vlaicu, M.; Constantin, S.; Știucă, E.; Taron, U.H.; Hofreiter, M.; et al. Late Pleistocene Occurrence of *Cuon alpinus* in Romania. In Proceedings of the Abstract Book; Lazăr, I., Grădinaru, M., Vasile, Ștefan, Eds.; Bucharest, 2017; p. 27.
28. Hublin, J.-J. The Modern Human Colonization of Western Eurasia: When and Where? *Quaternary Science Reviews* **2015**, *118*, 194–210, doi:10.1016/j.quascirev.2014.08.011.
29. Untergasser, A.; Nijveen, H.; Rao, X.; Bisseling, T.; Geurts, R.; Leunissen, J.A.M. Primer3Plus, an Enhanced Web Interface to Primer3. *Nucleic Acids Res* **2007**, *35*, W71–W74, doi:10.1093/nar/gkm306.
36. Martin, M. Cutadapt Removes Adapter Sequences from High-Throughput Sequencing Reads. *EMBnet.journal* **2011**, *17*, 10–12.
37. Magoč, T.; Salzberg, S.L. FLASH: Fast Length Adjustment of Short Reads to Improve Genome Assemblies. *Bioinformatics* **2011**, *27*, 2957–2963, doi:10.1093/bioinformatics/btr507.
39. Li, H.; Durbin, R. Fast and Accurate Short Read Alignment with Burrows–Wheeler Transform. *Bioinformatics* **2009**, *25*, 1754–1760, doi:10.1093/bioinformatics/btp324.
45. Jónsson, H.; Ginolhac, A.; Schubert, M.; Johnson, P.L.F.; Orlando, L. MapDamage2.0: Fast Approximate Bayesian Estimates of Ancient DNA Damage Parameters. *Bioinformatics* **2013**, *29*, 1682–1684, doi:10.1093/bioinformatics/btt193.
46. Urios, V.; Donat-Torres, M.P.; Ramírez, C.; Monroy-Vilchis, O.; Rgribi-Idrissi, H. El Análisis Del Genoma Mitocondrial Del Cánido Estudiado En Marruecos Manifiesta Que No Es Ni Lobo (*Canis lupus*) Ni Chacal Euroasiático (Canis Aureus). *Altoter0* **2015**, *3*, 2–15.
47. Björnerfeldt, S.; Webster, M.T.; Vilà, C. Relaxation of Selective Constraint on Dog Mitochondrial DNA Following Domestication. *Genome Res* **2006**, *16*, 990–994, doi:10.1101/gr.5117706.
48. Pang, J.-F.; Kluetsch, C.; Zou, X.-J.; Zhang, A.; Luo, L.-Y.; Angleby, H.; Ardalan, A.; Ekström, C.; Sköllermo, A.; Lundeberg, J.; et al. MtDNA Data Indicate a Single Origin for Dogs South of Yangtze River, Less than 16,300 Years Ago, from Numerous Wolves. *Mol. Biol. Evol.* **2009**, *26*, 2849–2864, doi:10.1093/molbev/msp195.
49. Thalmann, O.; Shapiro, B.; Cui, P.; Schuenemann, V.J.; Sawyer, S.K.; Greenfield, D.L.; Germonpré, M.B.; Sablin, M.V.; López-Giráldez, F.; Domingo-Roura, X.; et al. Complete Mitochondrial Genomes of Ancient

Canids Suggest a European Origin of Domestic Dogs. *Science* **2013**, *342*, 871–874, doi:10.1126/science.1243650.

50. Arnason, U.; Gullberg, A.; Janke, A.; Kullberg, M. Mitogenomic Analyses of Caniform Relationships. *Molecular Phylogenetics and Evolution* **2007**, *45*, 863–874, doi:10.1016/j.ympev.2007.06.019.
51. Campana, M.G.; Parker, L.D.; Hawkins, M.T.R.; Young, H.S.; Helgen, K.M.; Szykman Gunther, M.; Woodroffe, R.; Maldonado, J.E.; Fleischer, R.C. Genome Sequence, Population History, and Pelage Genetics of the Endangered African Wild Dog (*Lycaon pictus*). *BMC Genomics* **2016**, *17*, 1013, doi:10.1186/s12864-016-3368-9.
52. Kazuno, A.-A.; Munakata, K.; Mori, K.; Tanaka, M.; Nanko, S.; Kunugi, H.; Umekage, T.; Tochigi, M.; Kohda, K.; Sasaki, T.; et al. Mitochondrial DNA Sequence Analysis of Patients with 'Atypical Psychosis.' *Psychiatry and Clinical Neurosciences* **2005**, *59*, 497–503, doi:https://doi.org/10.1111/j.1440-1819.2005.01404.x.
53. Hofman, C.A.; Rick, T.C.; Hawkins, M.T.R.; Funk, W.C.; Ralls, K.; Boser, C.L.; Collins, P.W.; Coonan, T.; King, J.L.; Morrison, S.A.; et al. Mitochondrial Genomes Suggest Rapid Evolution of Dwarf California Channel Islands Foxes (*Urocyon littoralis*). *PLOS ONE* **2015**, *10*, e0118240, doi:10.1371/journal.pone.0118240.
56. Kumar, S.; Stecher, G.; Tamura, K. MEGA7: Molecular Evolutionary Genetics Analysis Version 7.0 for Bigger Datasets. *Mol. Biol. Evol.* **2016**, *33*, 1870–1874, doi:10.1093/molbev/msw054.
66. Robinson, J.T.; Thorvaldsdóttir, H.; Winckler, W.; Guttman, M.; Lander, E.S.; Getz, G.; Mesirov, J.P. Integrative Genomics Viewer. *Nat Biotechnol* **2011**, *29*, 24–26, doi:10.1038/nbt.1754.
83. Mallye, J.-B.; Costamagno, S.; Boudadi-Maligne, M.; Prucca, A.; Lauroulandie, C. Dhole (*Cuon alpinus*) as a bone accumulator and new taphonomy agent? the case of Noisetier cave (French Pyrenees). *Journal of Taphonomy* **2012**, *10*, 317–347.
84. Baryshnikov, G.F. Late Pleistocene Canidae Remains from Geographical Society Cave in the Russian Far East. *Russian Journal of Theriology* **2015**, *14*, 65–83.
85. Liu, W.; Wu, X.; Pei, S.; Wu, X.; Norton, C.J. Huanglong Cave: A Late Pleistocene Human Fossil Site in Hubei Province, China. *Quaternary International* **2010**, *211*, 29–41, doi:10.1016/j.quaint.2009.06.017.
86. Qinqi, X. Some remarks on Chenjiawo fauna-On the First Appearance of Peking Man Fauna. *Vertebrata Pal Asiatica* **1996**, *34*, 41–57.
87. Weiwen, H.; Xinqiang, S.; Yamei, H.; Miller-Antonio, S.; Schepartz, L.A. Excavations at Panxian Dadong, Guizhou Province, Southern China. *Current Anthropology* **1995**, *36*, 844–846, doi:10.1086/204441.
88. Suraprasit, K.; Jaeger, J.-J.; Chaimanee, Y.; Chavasseau, O.; Yamee, C.; Tian, P.; Panha, S. The Middle Pleistocene Vertebrate Fauna from Khok Sung (Nakhon Ratchasima, Thailand): Biochronological and Paleobiogeographical Implications. *Zookeys* **2016**, 1–157, doi:10.3897/zookeys.613.8309.
89. Long, V.T.; de Vos, J.; Ciochon, R.L. The Fossil Mammal Fauna of the Lang Trang Caves, Vietnam, Compared with Southeast Asian Fossil and Recent Mammal Faunas: The Geographic Implications. *The Bulletin of the Indo-Pacific Prehistory Association* **1996**, *14*, 101–109.

Publisher's Note: MDPI stays neutral with regard to jurisdictional claims in published maps and institutional affiliations.

© 2020 by the authors. Submitted for possible open access publication under the terms and conditions of the Creative Commons Attribution (CC BY) license (http://creativecommons.org/licenses/by/4.0/).
