# Supplementary figures and images for: Ancient DNA from the Asiatic Wild Dog (Cuon alpinus) from Europe
Source: Genes (Basel). 2021 Jan 22;12(2):144. doi: 10.3390/genes12020144 (PMC7911384; doi:10.3390/genes12020144)

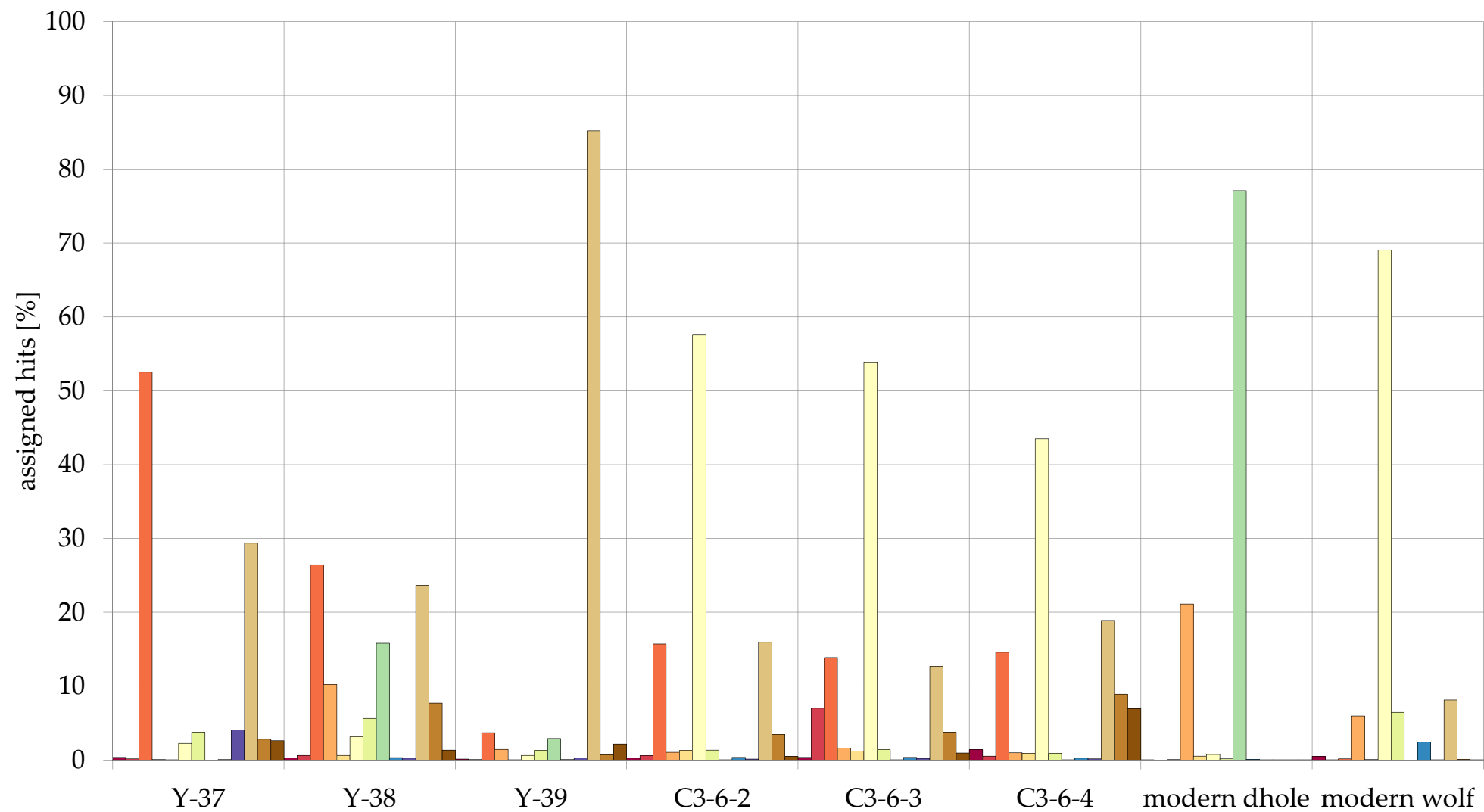

Supplement: Supplementary file 1 [file genes-12-00144-s001.zip › FigureS1_Blast_preprocessed_Reads.pdf]

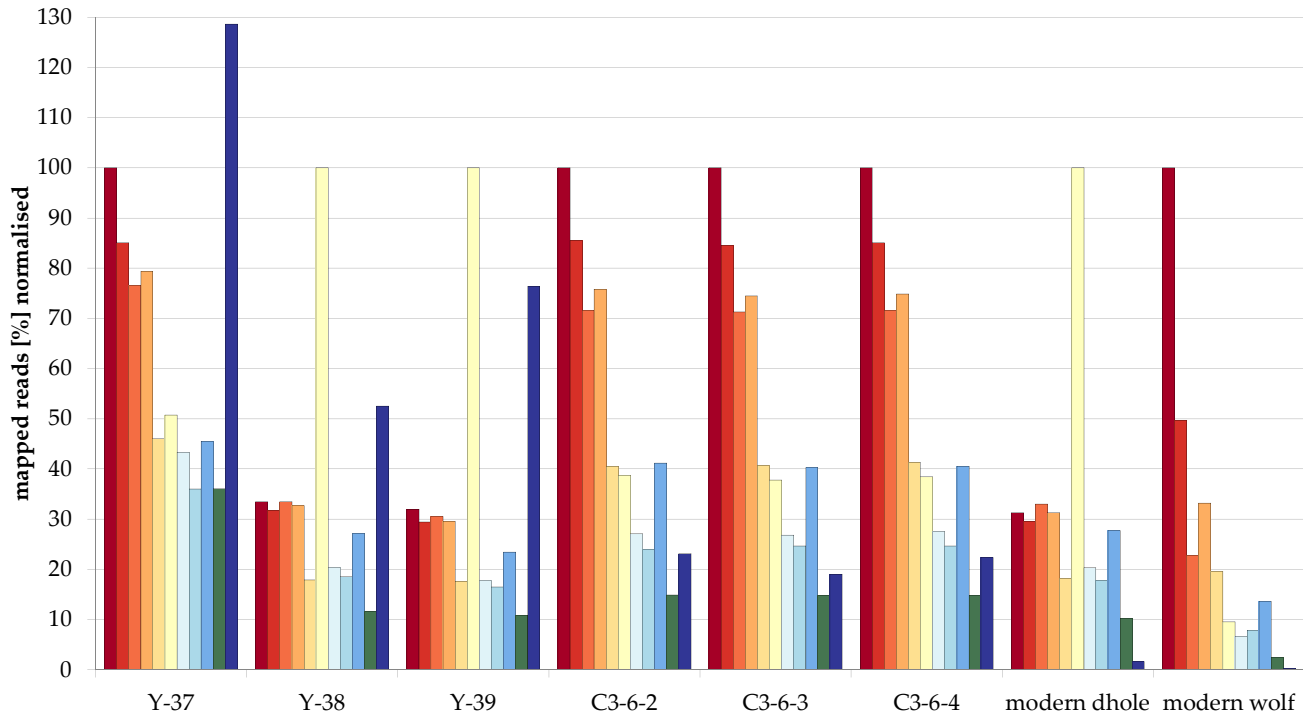

Supplement: Supplementary file 1 [file genes-12-00144-s001.zip › FigureS2_PercentageMappedReads_normalised_wHuman.pdf.pdf]

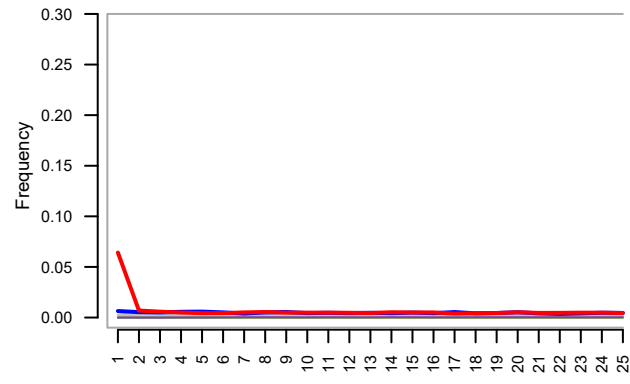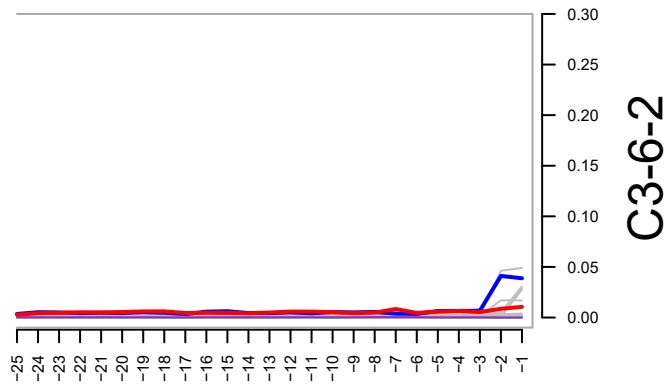

C3-6-2

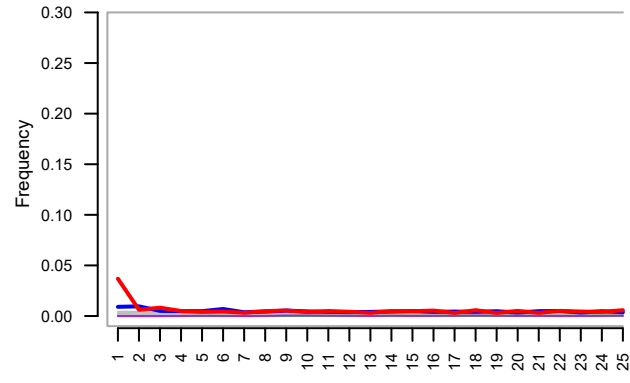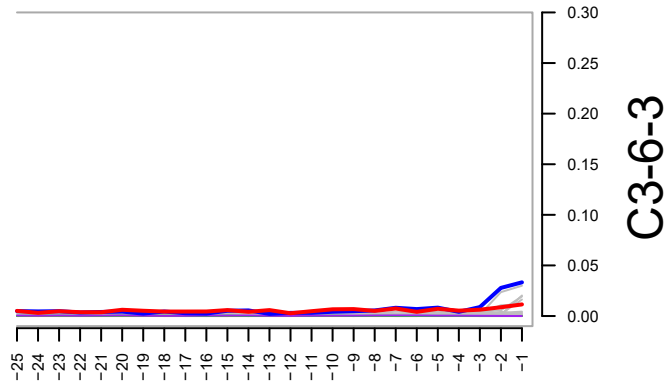

C3-6-3

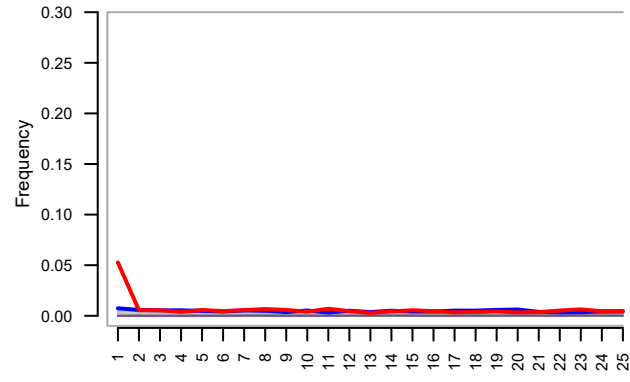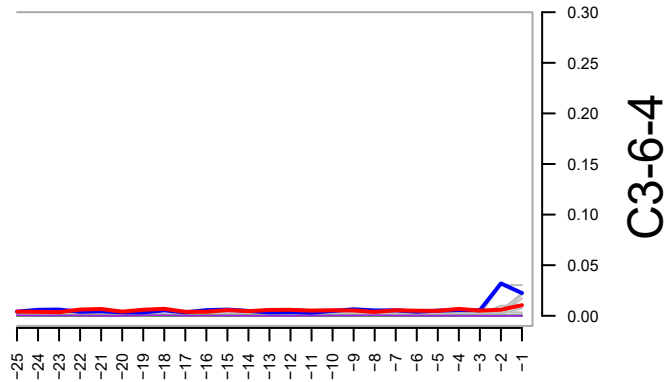

C3-6-4

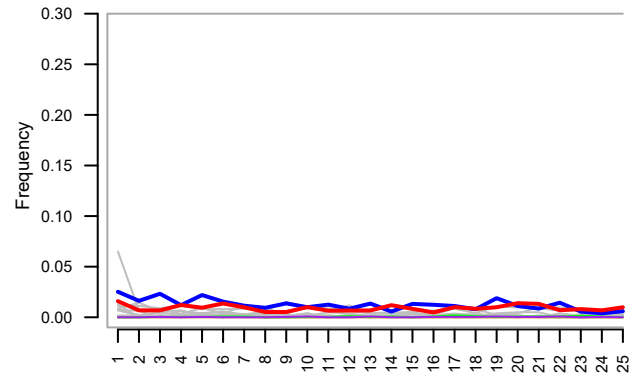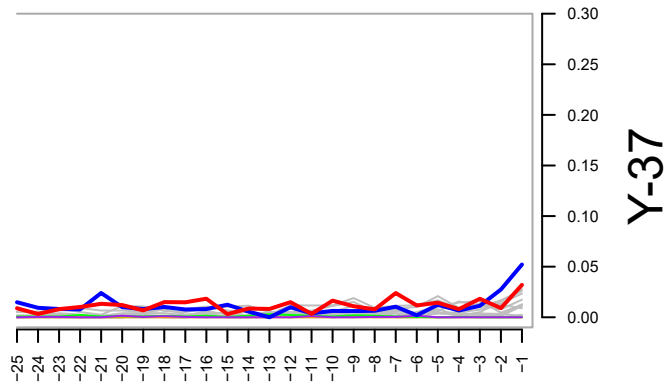

Y-37

Supplement: Supplementary file 1 [file genes-12-00144-s001.zip › FigureS3_mapDamage_C3-6-x_Y37.pdf]

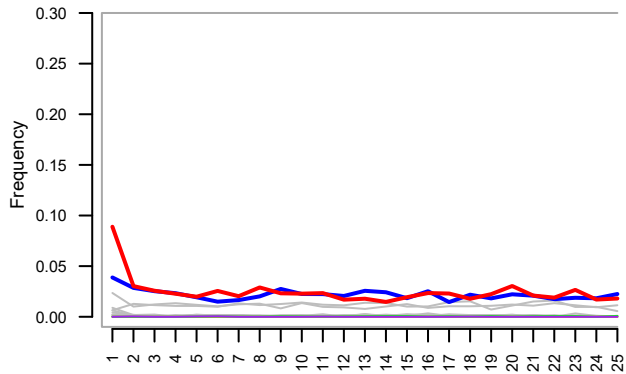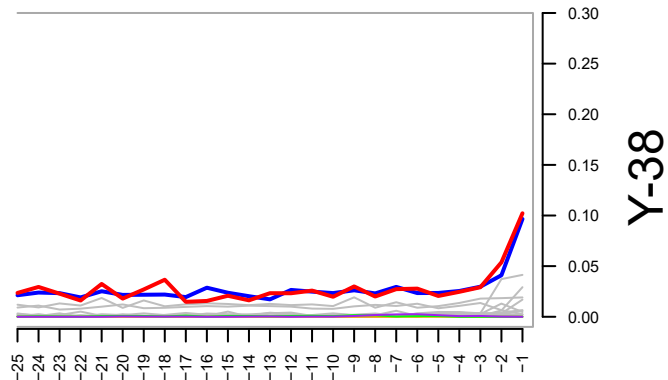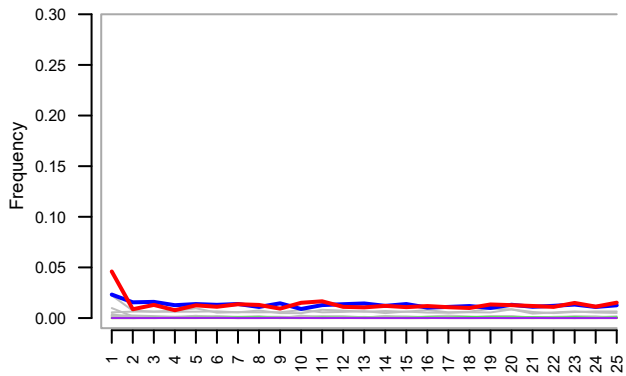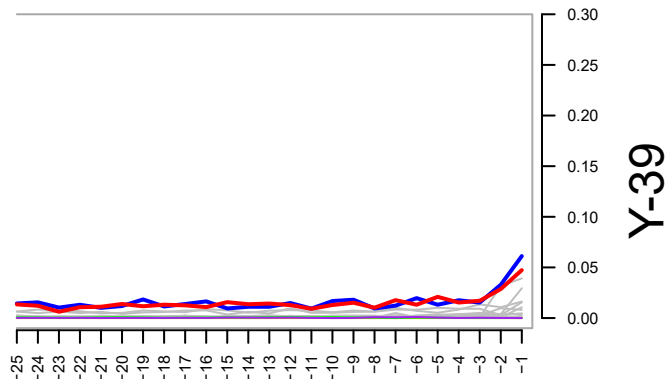

Supplement: Supplementary file 1 [file genes-12-00144-s001.zip › FigureS4_mapDamage_Y38_Y39.pdf]

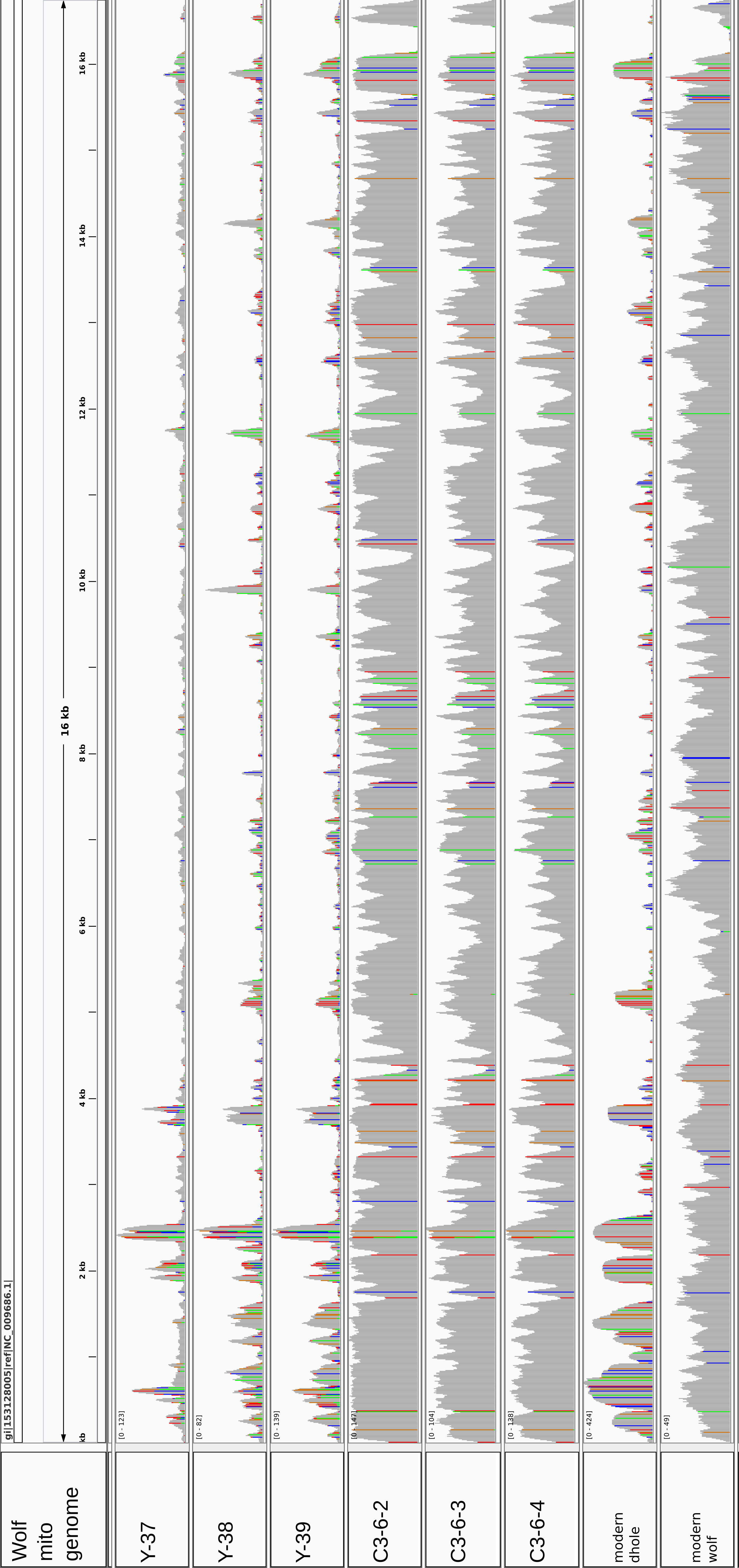

Supplement: Supplementary file 1 [file genes-12-00144-s001.zip › FigureS5_IGV_allSamplesWolf.png]

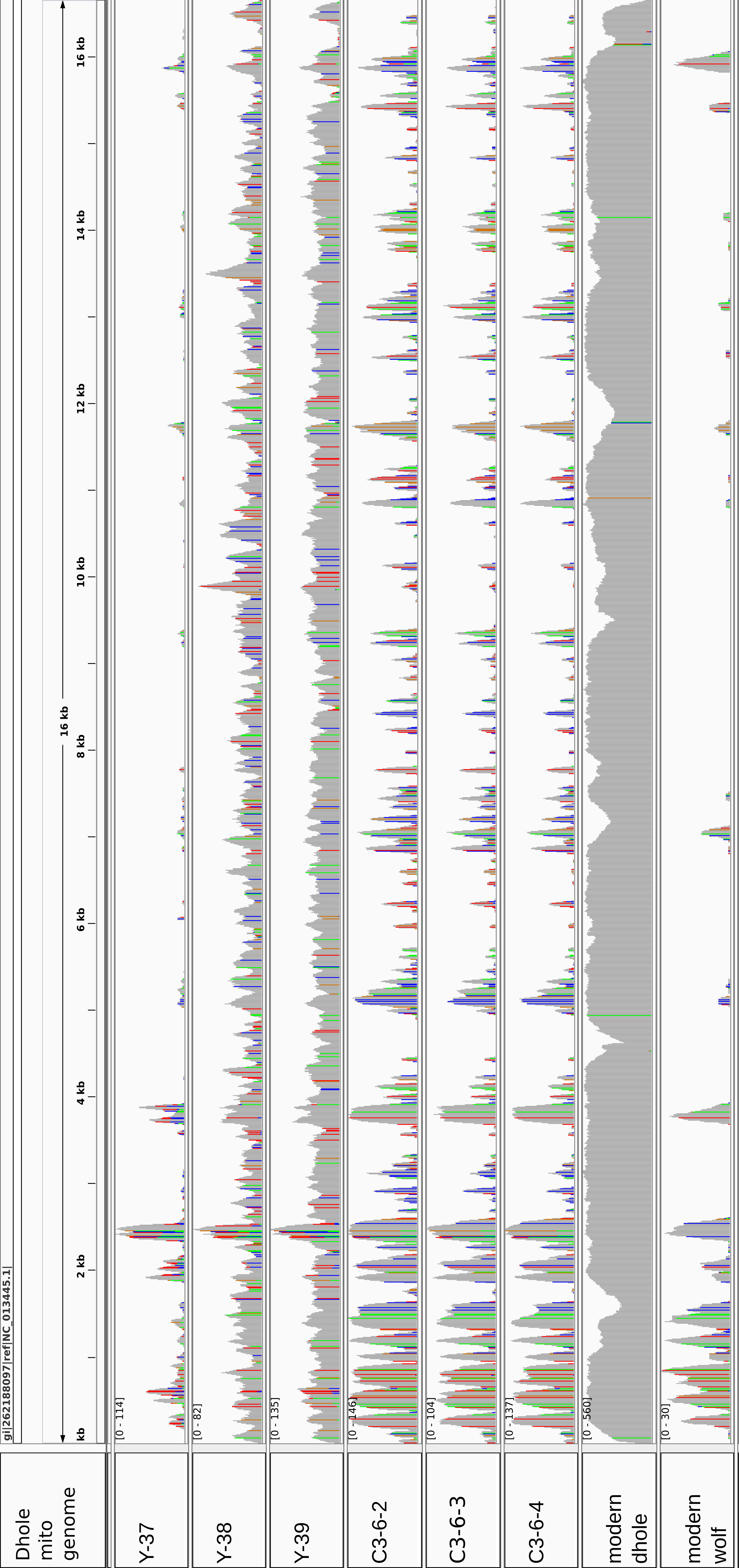

Supplement: Supplementary file 1 [file genes-12-00144-s001.zip › FigureS6_IGV_allSamplesCuon.png]

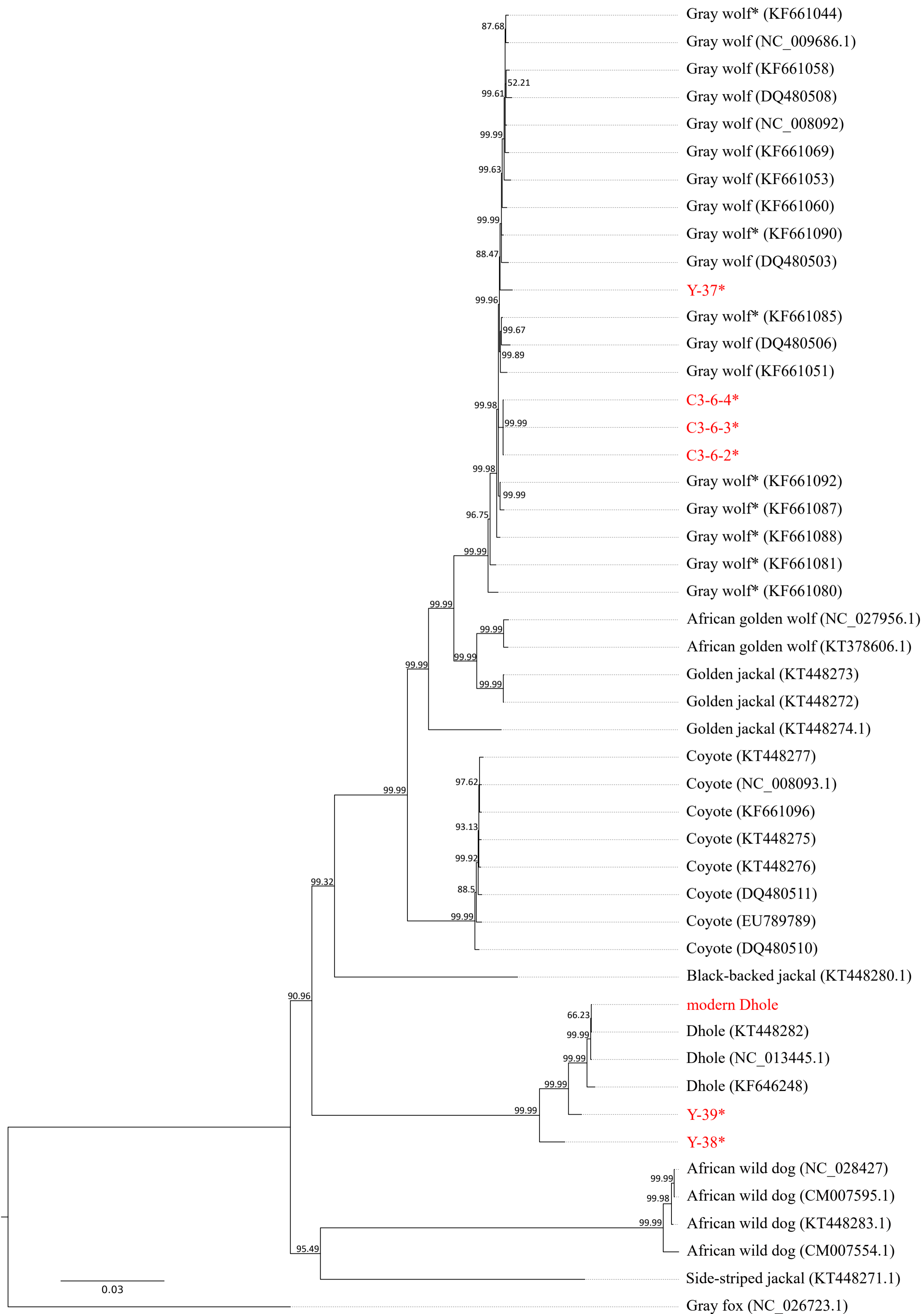

Supplement: Supplementary file 1 [file genes-12-00144-s001.zip › FigureS7_BayesianTree_supportTreshold50_BurnIn10.pdf]
